# Comparative Genomics of CytR, an Unusual Member of the LacI Family of Transcription Factors

**DOI:** 10.1371/journal.pone.0044194

**Published:** 2012-09-24

**Authors:** Natalia V. Sernova, Mikhail S. Gelfand

**Affiliations:** 1 A.A.Kharkevich Institute for Information Transmission Problems, Russian Academy of Sciences (IITP RAS), Moscow, Russia; 2 Faculty of Bioengineering and Bioinformatics, M.V.Lomonosov Moscow State University, Moscow, Russia; East Carolina University School of Medicine, United States of America

## Abstract

CytR is a transcription regulator from the LacI family, present in some gamma-proteobacteria including *Escherichia coli* and known not only for its cellular role, control of transport and utilization of nucleosides, but for a number of unusual structural properties. The present study addressed three related problems: structure of CytR-binding sites and motifs, their evolutionary conservation, and identification of new members of the CytR regulon. While the majority of CytR-binding sites are imperfect inverted repeats situated between binding sites for another transcription factor, CRP, other architectures were observed, in particular, direct repeats. While the similarity between sites for different genes in one genome is rather low, and hence the consensus motif is weak, there is high conservation of orthologous sites in different genomes (mainly in the Enterobacteriales) arguing for the presence of specific CytR-DNA contacts. On larger evolutionary distances candidate CytR sites may migrate but the approximate distance between flanking CRP sites tends to be conserved, which demonstrates that the overall structure of the CRP-CytR-DNA complex is gene-specific. The analysis yielded candidate CytR-binding sites for orthologs of known regulon members in less studied genomes of the Enterobacteriales and Vibrionales and identified a new candidate member of the CytR regulon, encoding a transporter named NupT (YcdZ).

## Introduction

CytR, the regulator of transport and utilization of nucleosides, was first mentioned in 1975 [1] and identified in 1985 [2]. The corresponding gene, *cytR*, was sequenced in 1986 [3].

Since then, nine *Escherichia coli* genes were experimentally shown to be regulated by CytR and form the CytR regulon: *deoC* [4], [5], *cytR* itself [6], *tsx* [7], *cdd* [8], *ppiA* [9], *nupC* [10], *nupG* [11], *udp* [12], *rpoH* [13]. Later the corresponding binding sites in the upstream regions of these genes, except *nupC*, were proposed. In other species, CytR was shown to regulate its own gene, *cytR*, in *Salmonella typhimurium* [14] and *udp* in *Salmonella typhimurium*, *Yersinia pestis* and *Vibrio cholerae* [15]. Since among the gamma-proteobacteria CytR is present only in *E. coli* and its close relatives (up to *Vibrionales*), it has been suggested that *cytR* appeared in the Enterobacteriales due to horizontal transfer from the delta-Proteobacteria (*Geobacillus* sp.) or *Caulobacter* sp. [16].

The structural and functional features of CytR were reviewed in [17]–[20]. CytR is an atypical representative of the LacI-family [21]. Its affinity to its operators is rather weak [22] and because of that, in contrast to most prokaryotic repressors, CytR alone is not capable to repress transcription. CytR functions in a complex with a multifunctional transcription factor (TF), CRP [23], [24].

The CRP protein is a dimer [25]. The subunit dimerization depends on the N-terminal domain, while the DNA recognition is performed by the C-terminal domain [26]. A possible regulatory mechanism was suggested, based on the crystal structure of CRP in complex with a DNA-fragment [27].

**Figure 1:**
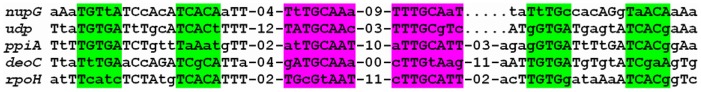
Organization of upstream regions of five experimentally proven *E. coli* members of the CytR regulon. [13], [19], [37]. CytR-binding sites (O_CYTR_) are highlighted in magenta, cores of CRP-binding sites (O_CRP_) – in green. Numbers denote spacer lengths. Dots denote gaps in the alignment.

CytR protein is also dimeric [28]. The number of CRP-binding sites (O_CRP_) per CytR-binding site (O_CYTR_) varies from one to three [17]: one, as in the *cytR* promoter [6]; two, as in the majority of cases; or three, as in the *cdd* promoter [8], [17], [29]. This might indicate different structures of the CRP-CytR complex or repositioning of the CRP dimers upon interaction. In most promoters, CRP has a stronger affinity to the distal operator O_CRP_D [30], with an exception being *cddP*, where CRP binds stronger to its proximal operator O_CRP_P [29]. An important requirement is that at least one CRP-operator has to be situated at a distance not exceeding 5 nucleotides to the corresponding CytR-operator [20], with the position of the O_CYTR_ operator being not symmetric relative to the flanking O_CRP_ operators [17]. Fig. 1 shows a typical organization of the O_CRP_D-O_CYTR_-O_CRP_P complex for five experimentally studied *E. coli* genes.

The mechanism of the CytR action is anti-activation rather than direct repression [17], [31], [32]. In particular, at the promoter *deo*P2, RNA polymerase and CytR compete for CRP that in this case acts as an activator [31]. CRP alone activates transcription, whereas the CRP-CytR cooperatively bound to O_CRP_ and O_CYTR_, respectively, represses transcription. Cytidine binding to CytR releases the latter from the complex, hence the activation by CRP resumes and the gene is derepressed [33], [34]. At that, the intrinsic CytR binding to DNA is not affected by cytidine binding [35]. The repression and activation of some other CytR-regulon genes were considered, in particular, in [17]. In the *cytR*, *deoP* and *udp* regulatory regions only one CRP-binding site participates in the activation; in the *nupG*, *tsx* and *cdd* promoters two CRP-binding sites are involved in the activation, and in all regulated genes, except *cytR*, two CRP-binding sites participate in the repression. Hence upstream regions of all CytR-regulated genes contain at least one CRP-binding site, either distal or proximal, that participates both in activation and repression, see [17] (Fig. 3, p.463).

The CytR-binding motif consists of two half-sites, denoted here as O_CYTR_D (distal) and O_CYTR_P (proximal). Unlike the situation with many TFs, including repressors from the LacI-family, the length of spacers between parts of the O_CYTR_ motif may vary in a wide interval from about zero to three DNA helical turns, with large spacers tending to comprise an integer number of turns, at most three [18].

In most studies, O_CYTR_D and O_CYTR_P were assumed to form degenerated inverted repeats [11], [13], [36], and the major role in specific binding was assigned to protein-protein (CytR-CRP) rather than protein-DNA (CytR-O_CYTR_) interactions. Still, at the physiological concentration of CytR, the CytR-DNA interactions are absolutely necessary for the repressor complex to be formed [37]. The exact O_CYTR_ position was mapped precisely in few cases only, e.g. in the *deo*P2 promoter by point mutagenesis [36] or by exchange of *udp*P and *deo*P2 O_CYTR_ operators [19]. In the majority of other cases, binding sites were located approximately by using the protein shift assay, protein footprinting with DNAase I or hydroxyl radical footprints, DMS-treatment, or cloning into a plasmid and measuring the level of CytR-repression [11], [22]. The exact position of O_CYTR_ was then predicted by the comparison with the consensus [11].

The latter was described as an inverted pentameric repeat TGCAA-N_2–3_-TTGCA [36], (where N denoted the number of nucleotides), a palindrome TTGCAA [38], or a pair of inverted octameric repeats (either 5′-AATGYCAAC-GC-GTTGCATT-3′ or 5′-AYGTGCAAC-N_x_-GTTRCATT-3′, where Y = T or C, R = A or G, and x = 10, 11, 12 or 13) which are the optimal CytR-binding sites in the absence of CRP, or direct repeats of octamers in either orientation with a 1 bp spacer [37]. The most recent description implies only octameric repeats with a spacer allowing both of them to be situated on the same side of the DNA-helix, with the spacer being less than 4–5 nucleotides or roughly a helical turn, that is 10–11 nucleotides [18], [20]. The current experimental data agree with this description [13], [29], [39]. The distances of about two or three helical turns were experimentally proven to be possible [18] but so far have not been observed in nature.

Here, we study the evolution of CytR-binding sites, characterize their common features, and identify new candidate members of the CytR regulon in the Enterobacteriales and Vibrionales.

## Results

### Recognition rules

**Figure 2:**
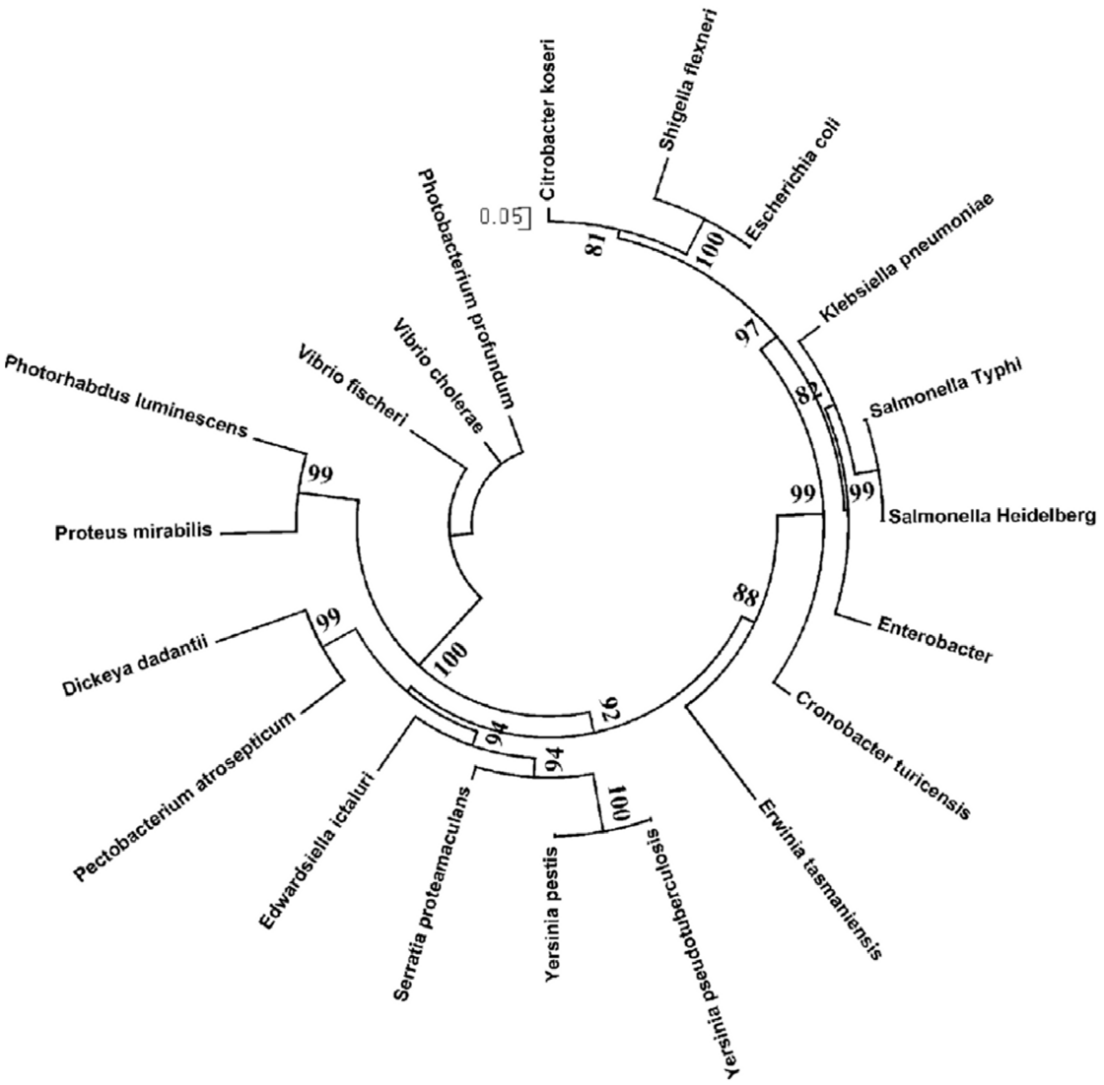
The ML-phylogenetic tree for the CytR proteins from the Enterobacteriales and Vibrionales. The tree defined the order of constructing alignments of upstream regions, see text for details.

We compiled a list of gamma-proteobacterial genomes encoding orthologs of CytR. Orthologs were initially defined by the bidirectional best hit criterion and confirmed by construction of phylogenetic trees (Fig. 2). All these genomes belong to the Enterobacteriales and Vibrionales, the list is given in Table 1. We also identified orthologs of genes known to be regulated by CytR in *E. coli* (Table S1) (see Data and Methods).

**Table 1:** The list of genomes with abbreviations.

3-letter abbreviation*	The full name of the bacteria	Accession number
CKO	*Citrobacter koseri*	NC_009792
CTU	*Cronobacter turicensis*	NC_013282
DDC	*Dickeya dadantii*	NC_013592
ECO	*Escherichia coli*	NC_000913
EIC	*Edwardsiella ictaluri*	NC_012779
ENT	*Enterobacter*	NC_009425
ETA	*Erwinia tasmaniensis*	NC_010693
KPE	*Klebsiella pneumoniae*	NC_011283
PEB	*Pectobacterium atrosepticum*	NC_004547
PLU	*Photorhabdus luminescens*	NC_005126
PMR	*Proteus mirabilis*	NC_010554
PPR	*Photobacterium profundum*	NC_006370(NC_006371)**
SEH	*Salmonella Heidelberg*	NC_011083
STY	*SalmonellaTyphi*	NC_003198
SFL	*Shigella flexneri*	NC_008258
SPE	*Serratia proteamaculans*	NC_009832
VCH***	*Vibrio cholerae*	NC_002505(NC_002506)
VFM***	*Vibrio fischeri*	NC_011184(NC_011186)
VHA***	*Vibrio harveyi*	NC_009783(NC_009784)
VPA***	*Vibrio parahaemolyticus*	NC_004603(NC_004605)
VSP***	*Vibrio splendidus*	NC_011753(NC_011744)
VVU***	*Vibrio vulnificus*	NC_004459(NC_004460)
YPN	*Yersinia pestis*	NC_008149
YPS	*Yersinia pseudotuberculosis*	NC_006155

* Abbreviations in the left column were taken from KEGG database.

** The accession number for the second chromosomes is in parentheses.

*** In the alignments, these genomes are denoted by the first two letters and a digit denoting the chromosome (1 or 2).

**Figure 3:**
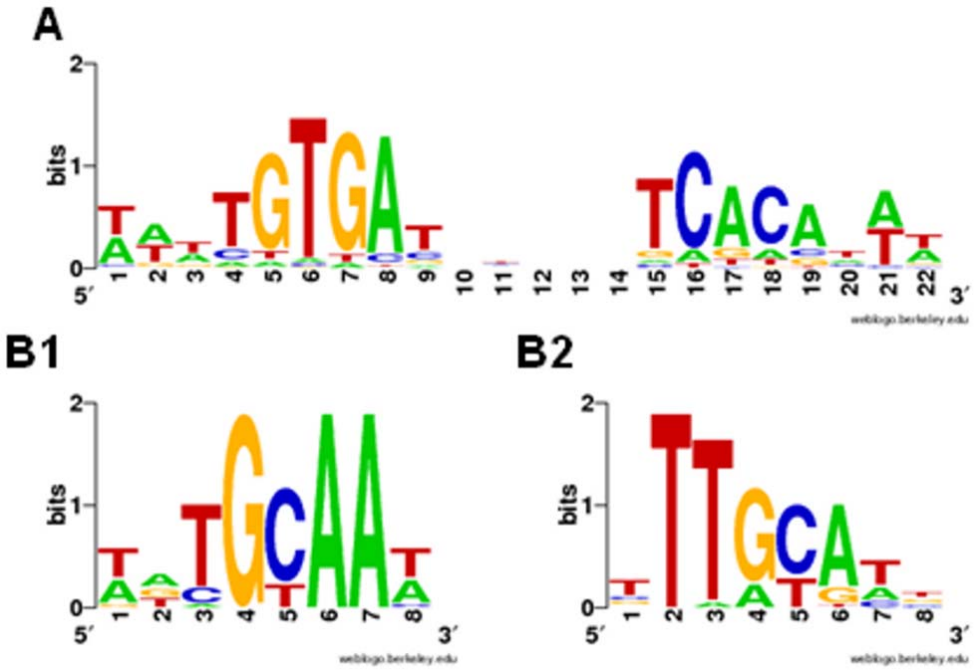
Sequence LOGOs of the CRP, CytR-distal, CytR-proximal operators. Horizontal axis: position in the binding site; vertical axis: informationcontent in bits. The height of each individual symbol reflects its prevalence at a given position, the height of each column is proportional to the positional information content in this position. A) O_CRP_ LOGO ; B1) O_CYTR_D LOGO; B2) O_CYTR_P LOGO.

**Figure 4:**
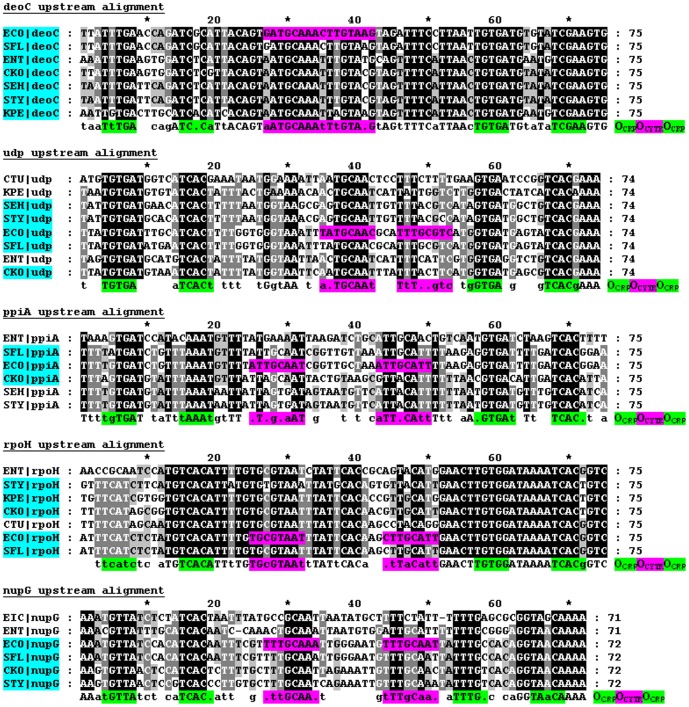
Alignments of upstream regions of gene orthologous to the *E.coli* CytR regulon members. O_CYTR_ boxes are highlighted in magenta. The consensus CytR and CRP motifs are shown at the bottom in magenta and green, respectively. Blue in the left column marks the genomes whose CytR-binding sites were used to construct the PWM. Shadows of grey denote the level of conservation, as set by GeneDoc: black – 100% conservation; dark gray – the consensus nucleotide frequency between 75% and 100%, light grey – the consensus nucleotide frequency between 50% and 75%; white – no conservation.

We used 69 published CRP-binding sites [40] to construct the O_CRP_ positional weight matrix (PWM) using SignalX (see Data and Methods).). Sequence LOGOs of the constructed motifs are shown in Fig. 3A. To construct the O_CYTR_ PWMs, we considered five *E. coli* genes with clearly distinguishable O_CYTR_ (Fig. 1). We performed multiple alignment of the upstream regions of these genes and their orthologs. At that, we gradually increased the number of aligned sequences, starting with closest *E. coli* relatives and then including more distant ones, in the order given by the phylogenetic tree of the CytR proteins (Fig. 2), while the O_CRP_ sites could be reliably aligned and the distance between them remained approximately constant. Then we selected only the sequences that were conserved in the regions corresponding to the *E. coli* sites: both O_CYTR_D and O_CYTR_P were taken for *deoC* from ECO, SFL, ENT, CKO, SEH, STY, KPE; for *udp* from SEH, STY, ECO, SFL, CKO; for *ppiA* from SFL, ECO, CKO; for *rpoH* from STY, KPE, CKO, ECO, SFL; and for *nupG* from ECO, SFL, CKO, STY; see Table 1 for genome abbreviations; the selected genes are highlighted in blue in Fig. 4. Sites in other species were accepted for the matrix construction if they satisfied the following conservation conditions: (1) the same distance between O_CRP_ sites for each *E. coli* gene listed above and its orthologs; (2) the same distance between O_CYTR_ half-operators; (3) at most two mismatches in each O_CRP_ site, and at most three total mismatches in the O_CRP_ sites, compared to the *E. coli* O_CRP_ sites; and (4) at most four mismatches in the O_CYTR_ operator, and at most three mismatches in each O_CYTR_ half-operator compared to the *E. coli* O_CYTR_ boxes. The selected boxes were used to construct PWMs for the upstream (distal) and downstream (proximal) half-operators (O_CYTR_D, Fig. 3B1, and O_CYTR_P, Fig. 3B2, respectively).

To identify new candidate CytR regulon members, we used three recognition rules to select regions for construction and manual analysis of alignments, requiring either (1) two candidate O_CRP_ sites at a distance 10–40 bp, or (2) two candidate O_CYTR_ sites at a distance not exceeding 20 bp, or (3) all four sites in the configuration O_CRP_D–N_(−10)–20_–O_CYTR_D–N_0–20_–O_CYTR_P–N_(−10)–20_–O_CRP_P, with negative numbers denoting overlapping sites.

For each set of orthologous genes, both known regulon members and new candidates, we performed multiple alignment anchored at pairs of the O_CRP_ operators. As mentioned above, in many genomes there are no strong candidate O_CYTR_ sites at positions corresponding to the *E. coli* O_CYTR_ operators. To identify possible shifted O_CYTR_ sites in the regions between pairs of O_CRP_ sites we used a variation of the sliding window technique, SWAS (sliding window average score) plots (see Data and Methods).

At each window position, we calculated the average weight of the O_CYTR_D and, separately, O_CYTR_P using respective PWMs. Our assumption was that if the position of the CytR-binding site O_CYTR_, comprising both O_CYTR_D and O_CYTR_P and the length of the spacer between them, was conserved within the alignment, the SWAS plot would have two pronounced peaks. On the other hand, if O_CYTR_D and/or O_CYTR_P shifted in a fraction of genomes, each new position would be represented by a new smaller peak. We accepted a peak if the average score within a window exceeded 3, or a single prominent peak with the score slightly below the average (e.g. about 2.7 for O_CYTR_ of *cdd* or *cytR*, see below). The positional conservation was also assessed using the plots of the information content (see Data and Methods). A SWAS peak was assumed to be more reliable if it was observed in a region of a more or less constant positional conservation.

### Evolution of CytR-binding sites

To characterize the conservation of CytR-binding sites, we constructed three groups of alignment of gene upstream regions for closest relatives of *E. coli*, for other Enterobacteriales (in some cases, for all available Enterobacteriales including *E .coli*), and for the Vibrionales, and analyzed these alignments using the SWAS plots. It should be noted that the representation of gene orthologs in genomes varied and, further, in some genomes the intergenic regions diverged beyond recognition. The criteria for the inclusion of upstream regions to alignments were based on the scores of the O_CRP_ sites and the conservation of the distance between them.

The operator cassettes may be classified into four main types by the pattern of conservation observed in the SWAS plots. The first type has two clear peaks that correspond to O_CYTR_D and O_CYTR_P, yielding conservation of both sites and the distance between them. The second rare type has one clear peak and a diffuse group of scattered minor peaks, reflecting conservation of one O_CYTR_ site and absence or shift of the other one. The third type is characterized by the absence of clear peaks. Finally, the fourth type is two peaks of the same type, reflecting direct rather than inverted repeats. There were few such cassettes, but they also could be conserved to some extent. Note that the above definitions may depend on the number and similarity of sequences in an alignment: the closer they are, the more likely the respective gene would belong to type 1 rather than to type 3.

**Figure 5:**
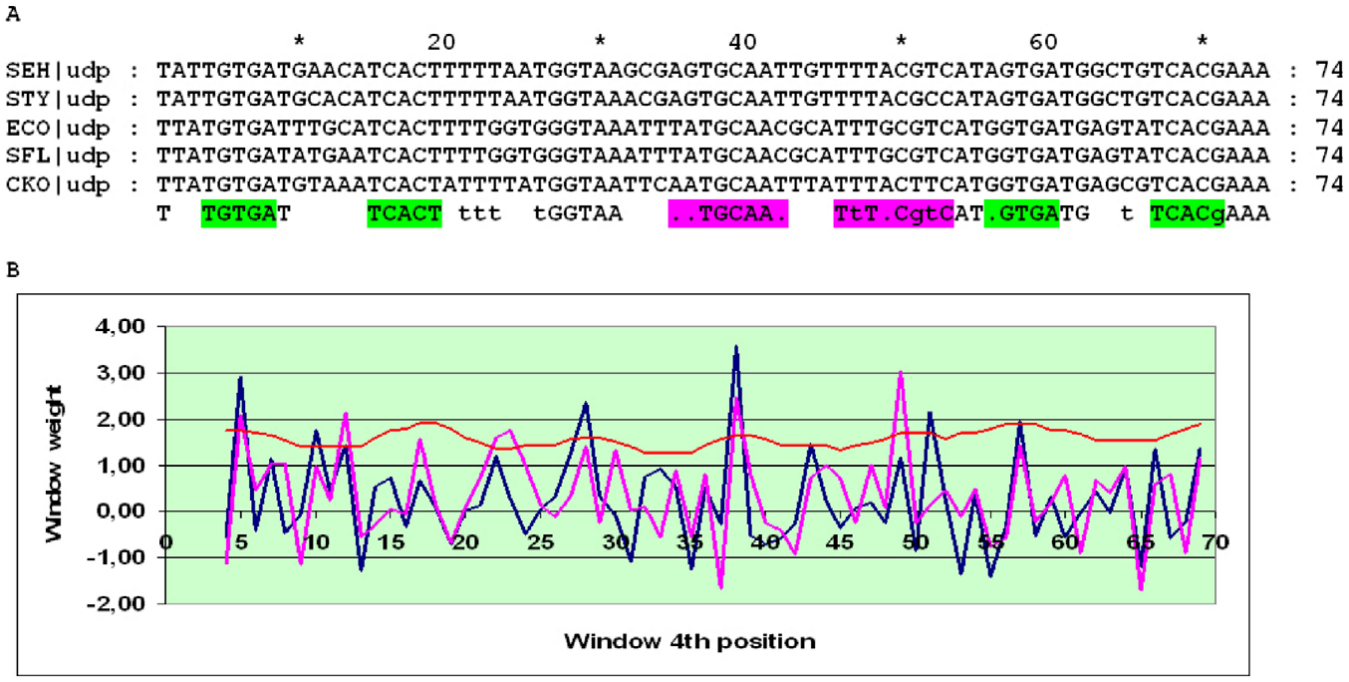
Alignment and SWAS plots of upstream regions of *udp* in close relatives of *E.coli*. The detected sites are highlighted in the consensus of alignment. A) Alignment of the upstream regions. Green – CRP-boxes, magenta – CytR-boxes. B) SWAS and information content plots. Scores are plotted corresponding to the middle (4^th^) position of a 8 bp window. Blue – O_CYTR_D, magenta – O_CYTR_P, red – averaged positional information content.

**Figure 6:**
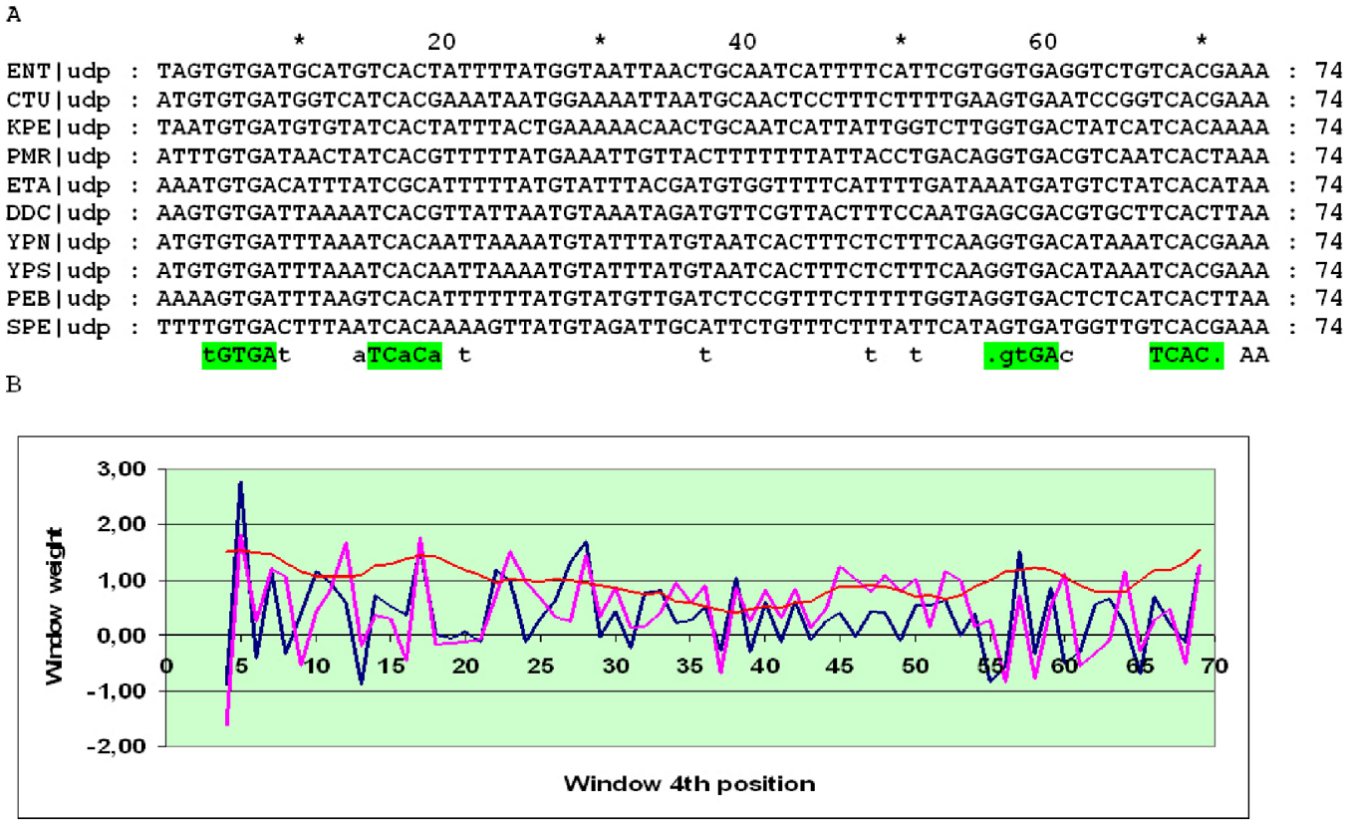
Alignment and SWAS plots of upstream regions of *udp* in distant Enterobacteriales. Notation as in Fig. 5.

**Figure 7:**
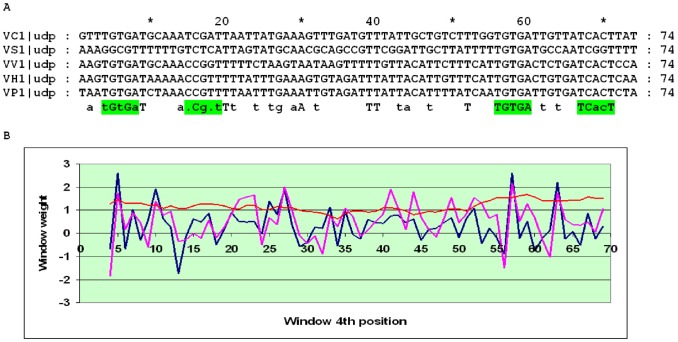
Alignment and SWAS plots of upstream regions of *udp* in the Vibrionales. Notation as in Fig. 5.

The *udp* gene encodes uridine phosphorylase in many Enterobacteriales and Vibrionales. The detailed structure of the *udp* cassette in *E. coli* was studied in [29]. The distance between the O_CRP_ sites in the *udp* promoter is conserved (30 or 31 bp) in almost all Enterobacteriales and Vibrionales; the only exceptions with non-conserved intersite distances and an overall low score of the cassette are *Photobacterium profundum*, *Photorhabdus luminescens* and *Vibrio fischeri*. The distances between the candidate O_CYTR_ sites are not constant, and the alignment may be divided into three subalignments. In the SWAS plot of close relatives of *E. coli*, two pronounced peaks corresponding to O_CYTR_D and O_CYTR_P are visible (Fig. 5). In more distant Enterobacteriales and in the Vibrionales, no clear peaks are seen, and there are many genome-specific non-conserved candidate O_CYTR_ sites, some overlapping with O_CRP_, that cannot be confidently predicted based on the sequence analysis alone (Fig. 6 and Fig. 7, respectively). Hence, the *udp* cassette is of type 1 at close distances and of type 3 at more distant ones.

The *deoC* gene encodes NAD(P)-linked 2-deoxyribose-5-phosphate aldolase. Prominent SWAS-plot peaks are observed in close relatives of *E. coli* where, unusually, there is no spacer between the O_CYTR_ sites: O_CYTR_D is immediately adjacent to O_CYTR_P (Fig. S1 and [29]). In more distant Enterobacteriales (*Edwardsiella ictaluri*, *Dickeya dadantii*, *Erwinia tasmaniensis* and *Klebsiella pneumoniae*) no clear peaks are seen (Fig. S2). In all Vibrionales including *P. profundum*, two *Yersinia* species (*Y. pestis* and *Y. pseudotuberculosis*) and *Pectobacterium atrosepticum*, the 4-box cassettes had very low total weights (about 10 or even less) and variable distances between the O_CRP_ sites, and hence the respective regions were not included into the alignments. Thus again we have type 1 behavior at close, and type 3 at distant Enterobacteriales.

Very low scores of 4-site cassettes were observed for most *ppiA* (peptidyl-prolyl cis-trans isomerase A) gene orthologs. Nevertheless, for six closest *E. coli* relatives, two peaks in the SWAS plot are clearly seen (Fig. S3), therefore the *ppiA* cassette is of type 1 at close Enterobacteriales, similar to previously characterized *ppiA* cassette in *E. coli*, see [9] (Fig. 1.B, p. 990).

The *rpoH* gene encodes the heat-shock sigma-factor (sigma-32 or σ^H^). The *E. coli* cassette was described in [13]. The 4-site cassette scores for this gene are rather low, mainly because of the low scores of the O_CRP_ sites. Moreover, the scores in most Enterobacteriales are lower than those in *E. coli*. However, the SWAS plot features two clear peaks (Fig. S4), thus the *rpoH* cassette belongs to type 1 in close *E. coli* relatives.

**Figure 8:**
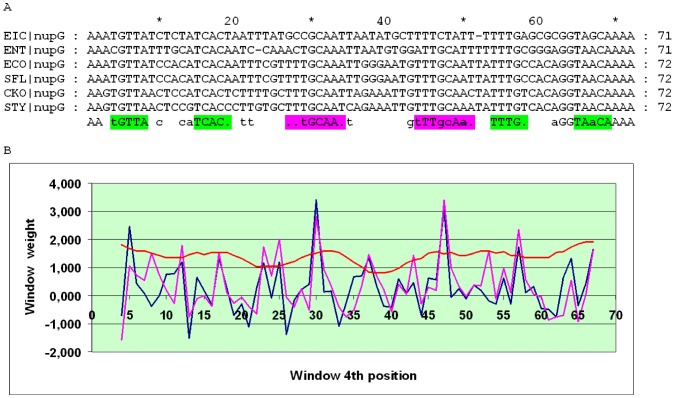
Alignment and SWAS plots of upstream regions of *nupG* in the Enterobacteriales. Notation as in Fig. 5.

The *nupG* gene encodes one of two high affinity nucleoside transporters in *E. coli*. It is present in seven genomes, the fewest among all considered genes. Further, the gene annotated as *nupG* in *Salmonella enterica* Heidelberg is in fact *xapB*, encoding xanthosine MFS transporter [41], as demonstrated by the analysis of phylogenetic trees (not shown) and co-localisation with *xapA*, the latter encoding a subunit of xanthosine phosphorylase. In *K. pneumoniae*, the total score of the best O_CRP_ pair is too low (about 7.1), and although the distance between them (30–31 nucleotides) is not sharply different from that in other genomes (27–28 bp), it is likely that the regulation of *nupG* in *K. pneumoniae* has been lost. The SWAS plot has two pronounced peaks corresponding to the O_CYTR_D and O_CYTR_P sites with the conserved distance of 9 bp between them and an overlap between the latter and the proximal O_CRP_ site (Fig. 8 and [39]). Hence the *nupG* cassette belongs to type 1.

**Figure 9:**
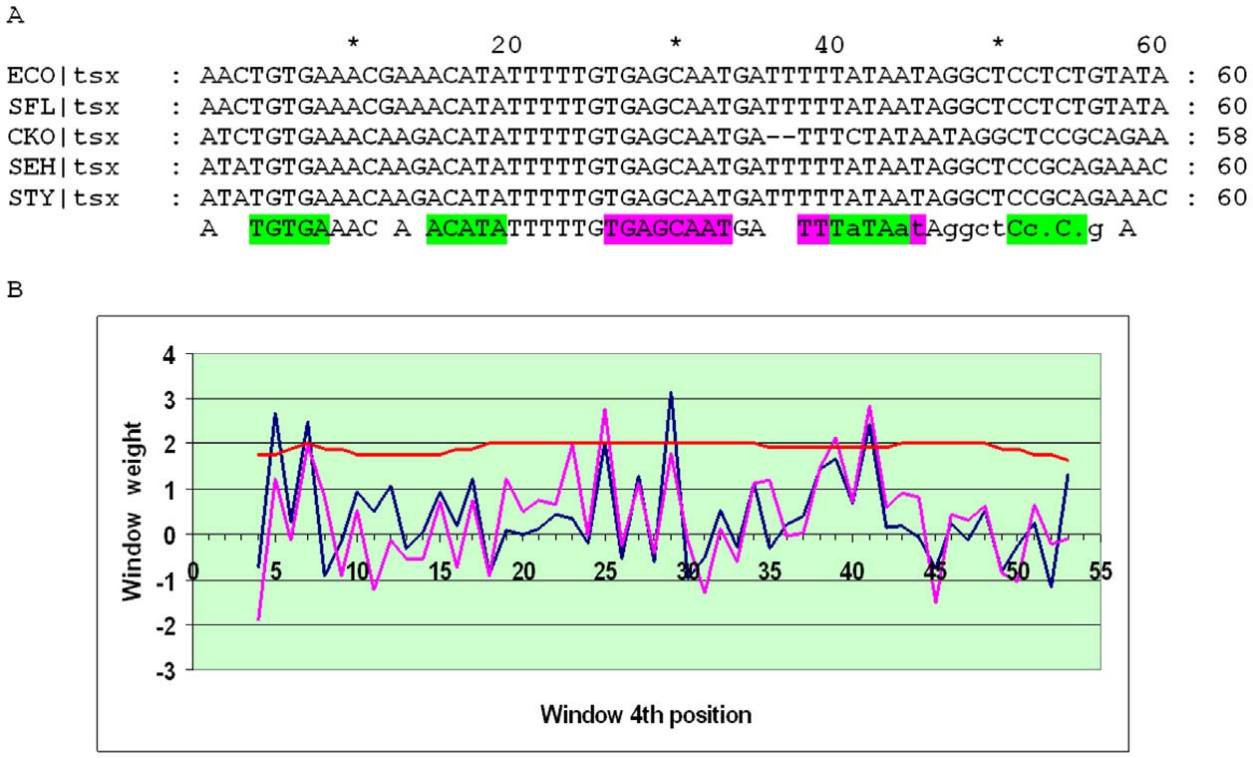
Alignment and SWAS plots of upstream regions of *tsx* in close relatives of *E. coli*. Notation as in Fig. 5.

**Figure 10:**
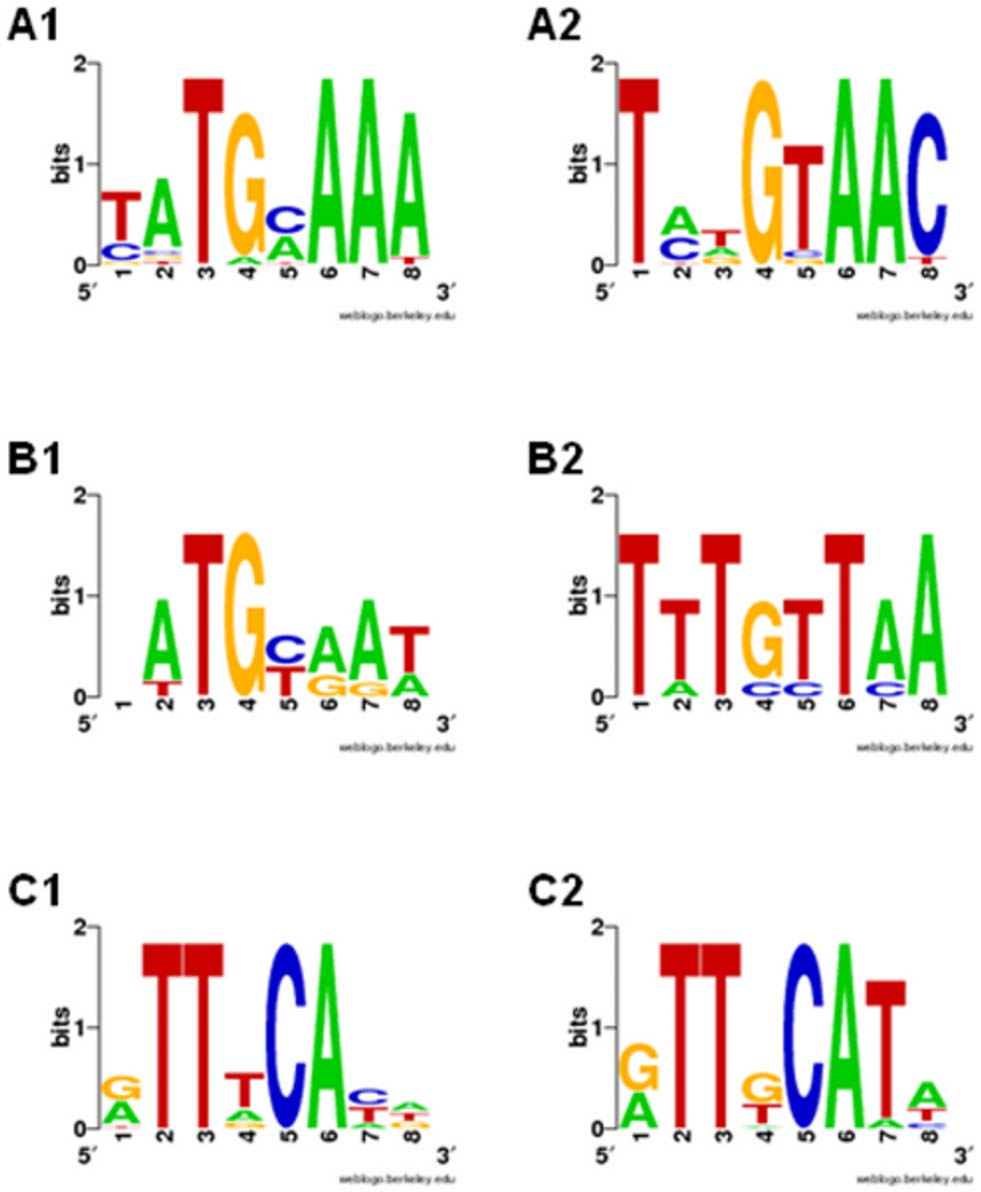
Sequence LOGOs of CytR-binding motifs, direct repeat type. Notation as in Fig. 3. A) O_CYTR_D LOGO for *cytR* from 16 Enterobacteriales; B) O_CYTR_D LOGO for *cytR* from 6 Vibrionales; C) O_CYTR_P LOGO for *cdd* from 14 Enterobacteriales.

The *tsx* gene, encoding the nucleoside channel, is present in many bacteria up to the Vibrionales. While the distance between the O_CRP_ sites flanking O_CytR_ is mostly conserved for the *tsx* orthologs in the Enterobacteriales, about 14 bp, the score of the 4-box cassette even in close relatives of *E. coli* is rather low, due to low scores of the O_CRP_ sites (about 3). However, the SWAS plot features two pronounced peaks, and hence the cassette is of type 1, although O_CYTR_P overlaps O_CRP_P (Fig. 9). The predicted sites in *E.coli* differ slightly from those suggested earlier (Fig. 9) here and [38] (Fig. 10, p.33253). Out of four other Enterobacteriales with *tsx* orthologs (*E. ictaluri*, *Enterobacter 638*, *K. pneumoniae*, *Serratia proteamaculans*) only three yield a relatively satisfactory alignment with the corresponding SWAS plot of type 2 (Fig. S5). Only four of the Vibrionales have *tsx* orthologs (*Vibrio harvey*, *Vibrio parahaemolyticus*, *Vibrio splendidus*, *Vibrio vulnificus*) (Fig. S6). Since a high-scoring O_CYTR_P peak is situated relatively close to O_CRP_D and all other peaks have very low scores (about 2), this case is assigned to type 3, as neither O_CYTR_D nor O_CRP_P can be reliably identified.

**Figure 11:**
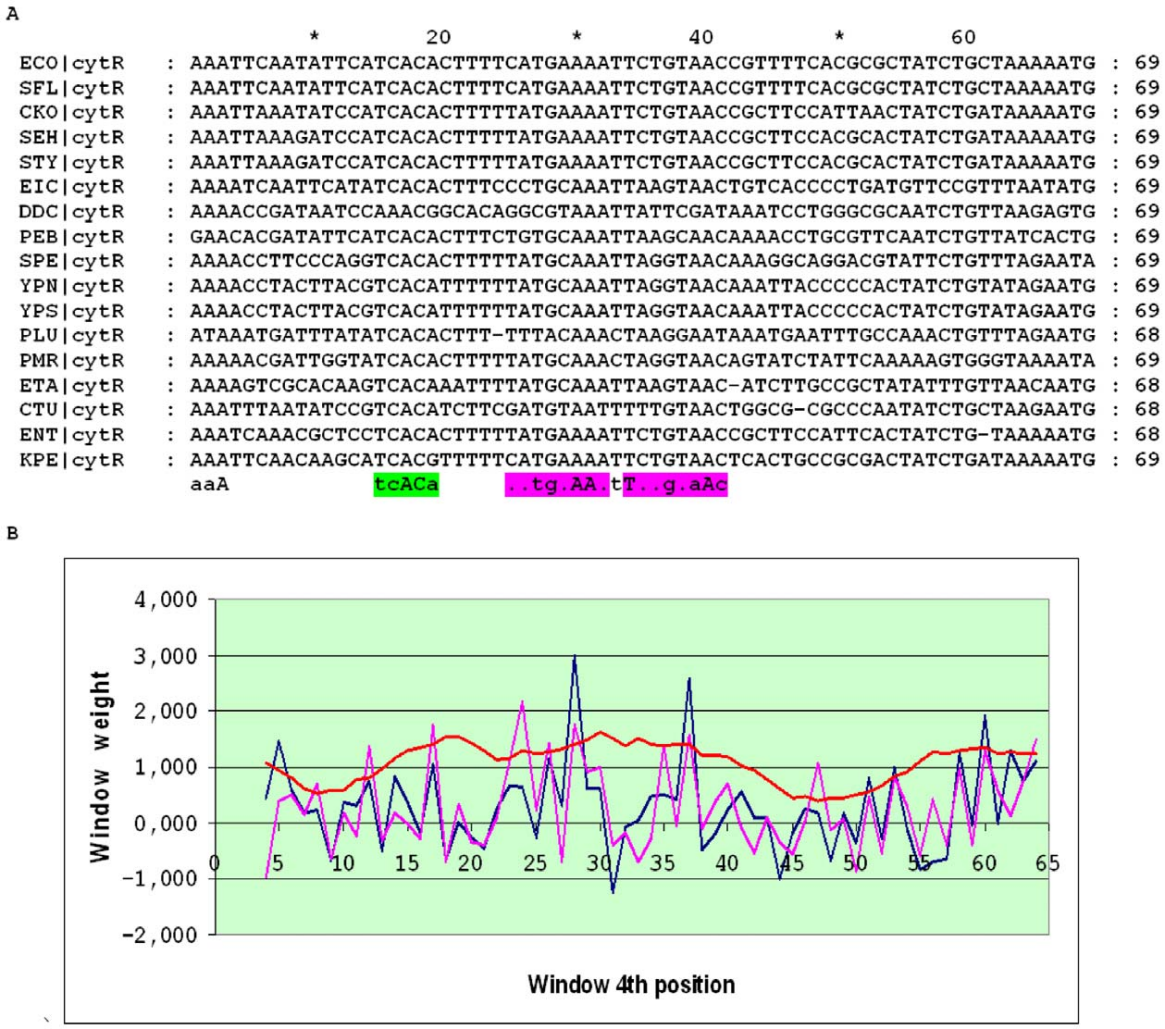
Alignment and SWAS plots of upstream regions of *cytR* in the Enterobacteriales. Notation as in Fig. 5.

**Figure 12:**
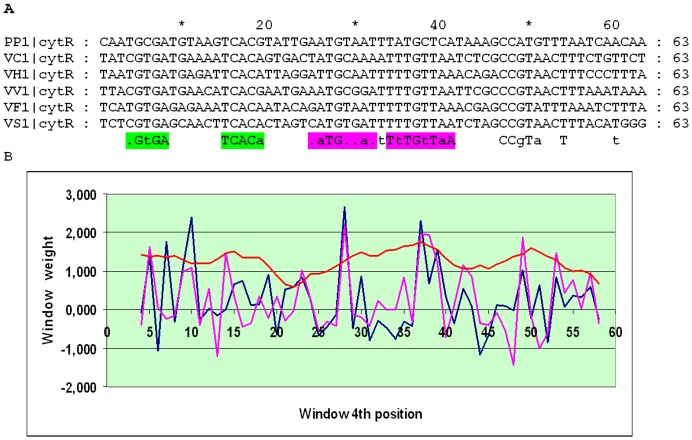
Alignment and SWAS plots of upstream regions of *cytR* in the Vibrionales. Notation as in Fig. 5.

The *cytR* gene itself has only the distal O_CRP_ site. On the other hand, the bound complex has been observed in *E. coli K-12* [6] and *S. typhimurium* [14]. The O_CYTR_ site is often assumed to be an imperfect inverted repeat [36], but the alignment of operator cassettes from sixteen Enterobacteriales and, separately, six Vibrionales shows that the O_CYTR_ site is an imperfect direct repeat (Fig. 10). At that, the Enterobacteriales and Vibrionales seem to have conserved organization of the O_CRP_D-O_CYTR_D-O_CYTR_P recognition site, but slightly different sequences of O_CYTR_D and O_CYTR_P. The unusual properties of this cassette may explain the fact that the scores of the O_CYTR_ sites are low, less than 3. However, the conservation of these sites in the alignment provides the evidence for their functional relevance (Fig. 11 and Fig. 12).

Another atypical cassette is that of *cdd*, cytidine/deoxycytidine deaminase. It contains O_CRP_ sites flanking direct repeats of O_CYTR_ [8], [29]. Two O_CRP_D sites denoted in the literature and here, in this particular case, O_CRP_2 and O_CRP_3 overlap by 20 bp, that is, they are shifted relative to each other by 2 bp. The arrangement of the sites is conserved in 14 genomes (Fig. S7 and Fig. S8).

To analyze direct repeats in the two latter cases, belonging to type 4, we applied the standard matrices for O_CYTR_D and O_CYTR_P (Fig. 3B1 and Fig. 3B2, respectively) and selected the matrix providing two highest SWAS-plot peaks. Both site cassettes have 1 bp spacers.

In the two cases of direct repeats, for the *cytR* cassette, pronounced SWAS-plot peaks were observed for the O_CYTR_D PWM both for the Enterobacteriales and Vibrionales (Fig. 11 and Fig. 12, respectively), whether for the *cdd* cassette visible peaks are produced by O_CYTR_P PWM for the Enterobacteriales, whereas none of the two matrices provides anything definite for the Vibrionales (Fig. S7 and Fig. S8, respectively).

**Figure 13:**
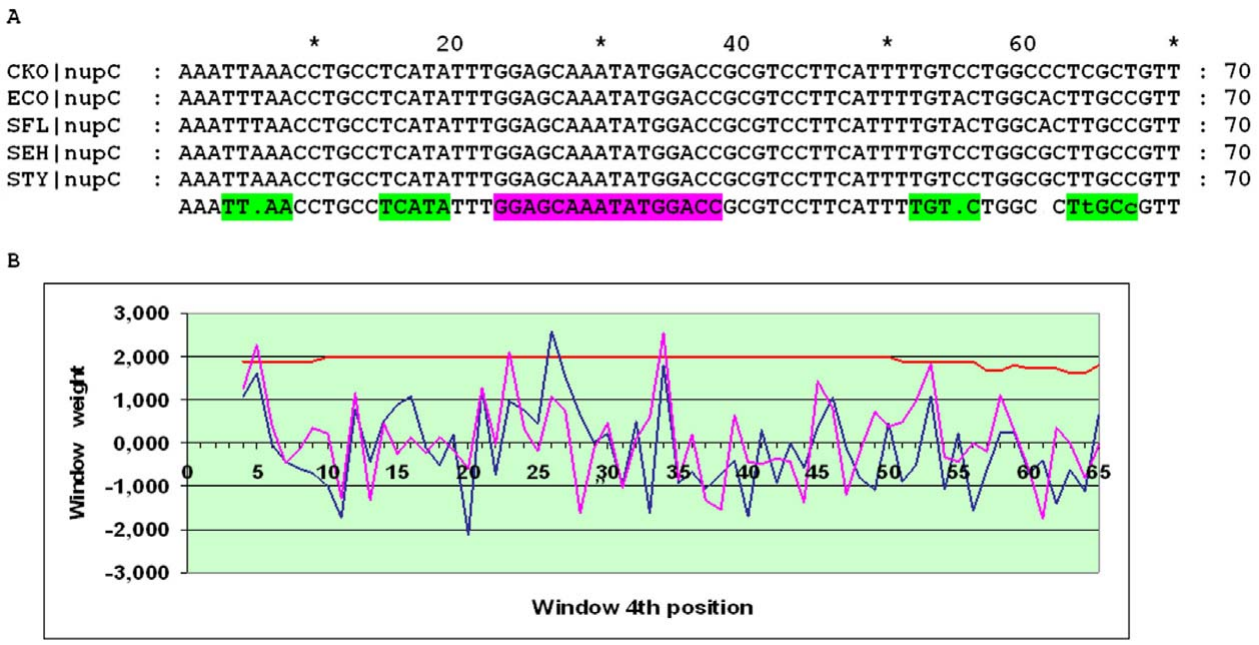
Alignment and SWAS plots of upstream regions of *nupC* in close relatives of *E. coli* (inverted O_CYTR_ repeats). Notation as in Fig. 5.

NupC is a nucleoside transporter. It is unrelated to NupG and shows somewhat different specificity: unlike universal NupG, it does not transport guanosine and deoxyguanosine [42]. The *nupC* gene was proposed to be regulated by CytR based on its function in the nucleoside transport, similar to some other genes from the CytR regulon [43], and the location of candidate pentameric binding sites [10]. While the alignment of the *nupC* upstream regions of five closest relatives of *E. coli* contains conserved regions, they have very low O_CRP_ scores. Candidate O_CYTR_ sites are seen in the alignment as inverted repeats at a zero distance (Fig. 13). The corresponding peaks at the SWAS plot are weak (∼2.6) but clearly visible. The score of O_CRP_D is about 4, which is consistent with a usual model of regulation of promoters with two CRP-binding sites. An alternative is O_CYTR_ being a direct repeat with a 3 bp spacer, close to the one observed in a SELEX experiment for direct repeats [37]. In this case the score of one of the peaks is larger than 3 and the score of the second peak is about 2.5, both assessed by the O_CYTR_P PWM (Fig. S9). Finally, there is a possibility that weaker O_CRP_ sites, in particular the one overlapping the transcription start site, also participate in formation of the regulatory complex. An experiment is needed to validate the predicted site and to select between the alternative descriptions of the cassette structure.

### New candidate member of the CytR regulon

As the initial criterion for identification of new possible operator cassettes, we relied on conservation of the distance between candidate O_CRP_ sites and the presence of peaks in the SWAS plots, demonstrating conservation of the O_CYTR_ positions. We started with identification of *E. coli* genes preceded by high-scoring O_CRP_D-O_CYTR_D–O_CYTR_P–O_CRP_P cassettes. We required that the score of each cassette exceeded the minimal observed score for the known genes (cut-off 12.6) and that the distance between O_CRP_ sites was in the interval (10–40). As expected, the initial four genes used to construct the PWMs (*deoC*, *nupG*, *ppiA*, *udp*) had high total scores and were among the leaders in the list ordered by decrease of the total O_CRP_-O_cytR_D-O_cytR_P-O_CRP_ score. We selected 37 *E.coli* genes satisfying these criteria, listed in Table S2.

Then we identified orthologs of these genes and checked the presence of a pair of O_CRP_ sites at approximately the same distance in at least five genomes. After that we aligned the promoter regions, anchored at O_CRP_D and O_CRP_P, and applied the O_CYTR_D and O_CYTR_P PWMs, constructing SWAS plots for the spacer between O_CRP_D and O_CRP_P.

One strong candidate emerged from this analysis. The *ycdZ* gene of *E .coli* is preceded by a cassette formed by two O_CRP_ sites at a conserved distance (29–31 bp) and O_CYTR_ sites in the correct arrangement, and this cassette is conserved in 17 related genomes, that is, in almost all Enterobacteriales and Vibrionales. The exceptions were *D. dadantii*, *E. tasmaniensis*, *P. atrosepticum*, *Y, pestis* and *Yersinia pseudotuberculosis*, where this gene is simply absent, and *V. vulnificus* that has an atypical O_CRP_–O_CRP_ distance.

**Figure 14:**
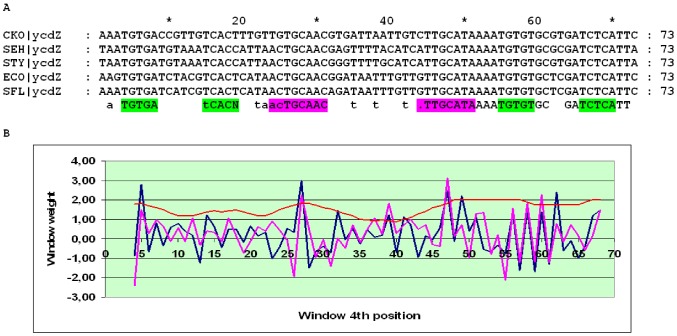
Alignment and SWAS plots of upstream regions of *ycdZ* in close relatives of *E. coli*. Notation as in Fig. 5.

**Figure 15:**
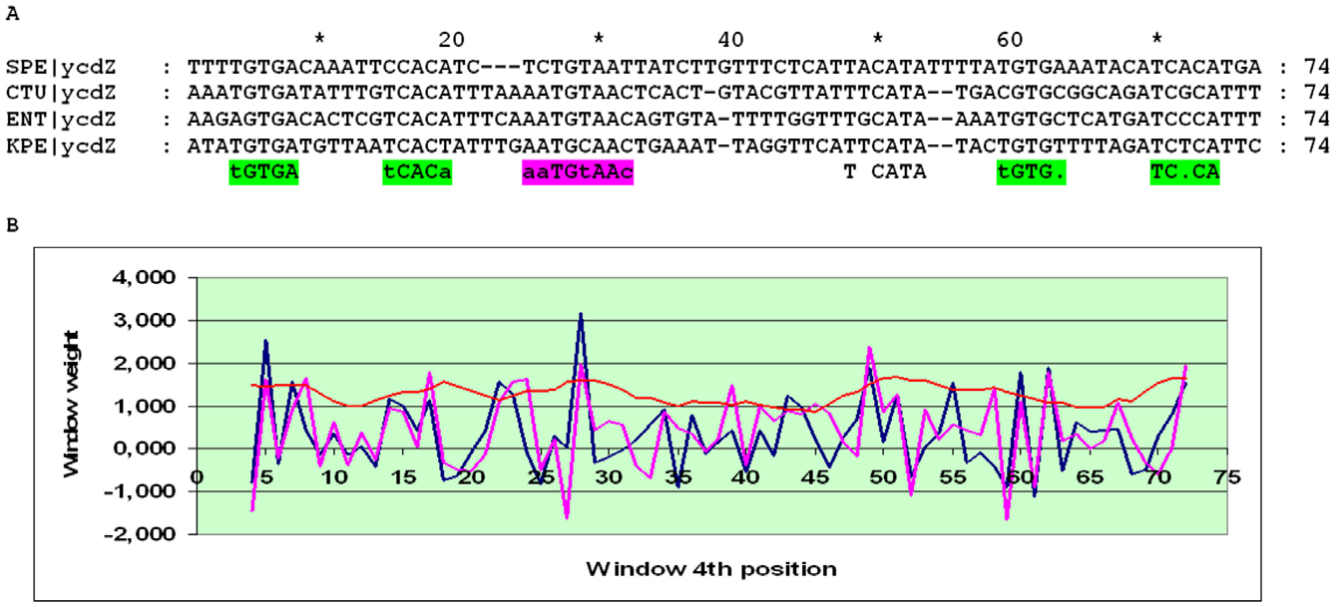
Alignment and SWAS plots of upstream regions of *ycdZ* in distant Enterobacteriales. Notation as in Fig. 5.

**Figure 16:**
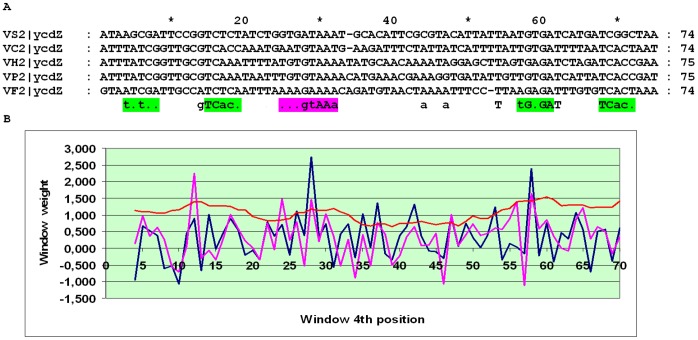
Alignment and SWAS plots of upstream regions of *ycdZ* in the Vibrionales. Notation as in Fig. 5.

The alignment of the *ycdZ* upstream regions may be divided into three subalignments. In close relatives of *E. coli*, two pronounced peaks in the SWAS plots, corresponding to O_CYTR_D and O_CYTR_P, are visible (Fig. 14). In more distant Enterobacteriales, one clear peak is seen (Fig. 15). In the Vibrionales, one peak is visible, but its average score is less than 3 (Fig. 16). Thus, the *ycdZ* cassette belongs to type 1 at close distances and to type 2 in more distant Enterobacteriales and in the Vibrionales. Hence we predict that *ycdZ* is a member of the CytR regulon.

**Figure 17:**
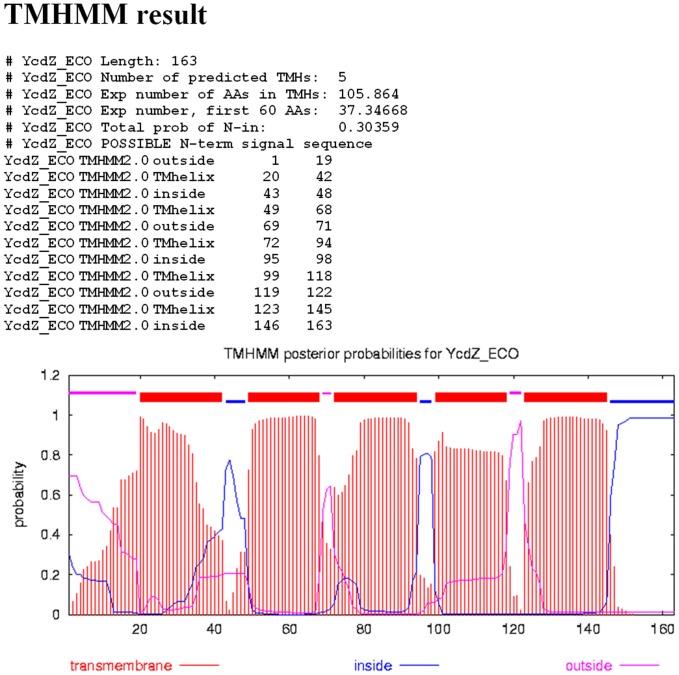
TMHMM predictions for YcdZ. Five transmembrane domains are predicted by TMHMM.

The encoded protein YcdZ is an inner-membrane protein from the DUF1097 family. According to TMHMM (see Data and Methods) it has five transmembrane domains (Fig. 17). Hence YcdZ is likely to be a transporter. We suggest naming it NupT.

## Discussion

**Figure 18:**
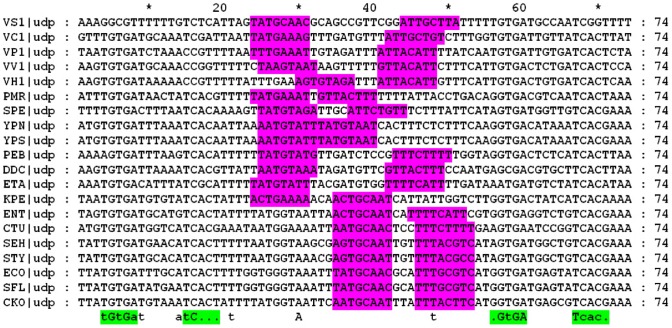
Alignment of upstream regions of *udp* with predicted O_CYTR_ sites, flanked by O_CRP_ sites in the Enterobacteriales and Vibrionales. Notation as in Fig. 5.

We have observed that the distances between candidate O_CRP_ sites are conserved in upstream regions of orthologous genes regulated by the CRP-CytR complex. On the other hand, positions of the O_CYTR_ sites seem to be conserved only at close evolutionary distances, as the highest-scoring candidate sites may occupy different positions in distant Enterobacteriales and in the Vibrionales (e.g. Fig. 18). One possible explanation for that, discussed in the literature, is that the binding of CytR to DNA has very low specificity, and the regulation is based on the formation of multimetric CRP-CytR complexes stabilized by the CytR-DNA interaction [44]. However, the existence of the CytR-binding motif, albeit weak, as well as the intergenome conservation (higher than background) of O_CYTR_ sites argues against this explanation. This is represented by peaks in the SWAS plots.

On the other hand, the intergenome conservation of the distances between O_CRP_ sites in promoters of specific genes together with intragenome differences between genes and relatively low conservation of positions of candidate O_CYTR_ sites demonstrate that the structure of the complex is dictated by CRP molecules.

The problem of identification of the CytR-binding motif is not trivial either. Indeed, the experimental data do not define the binding sites up to nucleotide: the most commonly used method, DNA footprinting, leaves some uncertainty about the site extent and location [45]. When the motif is strong, it is simple to align the footprinted regions and identify the common core. However, for weak motifs this is far from being straightforward, and we believe that evolutionary considerations yielding the phylogenetic footprinting techniques also deserve attention.

Finally, the overall structure of the CytR-binding site may vary. In most cases, it is an inverted repeat with a variable spacer. However, as shown in a SELEX experiment with the *deo* operator, both inverted repeats of O_CYTR_ boxes with a large spacer (10 to 13 bp) and direct repeats in either direction (O_CYTR_D or O_CYTR_P) with a short spacer (1 bp) may be bound by CytR [37]. Direct repeats in the O_CYTR_D orientation were observed to be conserved in the operators of *cytR* and *cdd*, again, with short spacers.

The comparative analysis also enables to identify new regulon members even for regulators with weak motifs. Of course, the predicted CytR regulation of the *nupT (ycdZ)* gene requires experimental verification.

## Data and Methods

Complete genome sequences of the Enterobacteriales and Vibrionales in the gbk format were downloaded from GenBank (ftp://ftp.ncbi.nih.gov/genomes/Bacteria) [46].

Multiple alignments of protein and DNA sequences were constructed using Muscle 3.6 (http://www.drive5.com/muscle/) [47] and visualized and manually edited using GeneDoc Editor version 2.6.002 (http://www.nrbsc.org/gfx/genedoc/; Nicholas, Karl B and Nicholas, Hugh B. Jr. 1997, GeneDoc: a tool for editing and annotating multiple sequence alignments. Distributed by the authors). Protein sequence database searches were performed using the latest version of BLASTP (ftp://ftp.ncbi.nlm.nih.gov/blast/executables/blast/2.2.25) [48], . All searches were run against the non-redundant protein sequence database at the NCBI. Positional information content of nucleotide alignments was calculated using ad hoc programs. Transmembrane segments were identified by TMHMM (http://www.cbs.dtu.dk/services/TMHMM/) [49] and candidate N-terminal signal sequences were analyzed with SWMSignal (http://bio-cluster.iis.sinica.edu.tw/SVMSignal) [50].

The maximum-likelihood (ML) tree was constructed using morePHYML, (http://mobyle.pasteur.fr/cgi-bin/portal.py?%23forms::phyml#forms::morePhyML) [51]. The circular tree was built by MEGA 5.1 [52].

Motif identification, construction of recognition profiles, identification of candidate sites in genome sequences and protein similarity searches using the Smith–Waterman algorithm were performed using Genome Explorer, version 3 [53], modified by L.V. Lunovskaya and A. Shpilman. Candidate sites were identified in the interval (−300,+200) relative to the start codon. Positional weight matrices were constructed using SignalX (http://bioinf.fbb.msu.ru/SignalX) [54]. Sequence logos were constructed by Web-LOGO (http://weblogo.berkeley.edu/logo.cgi) [55] .

The Positional Weight Matrix (PWM) was defined via:
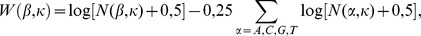
where *W*(*β*,*κ*) is the positional weight of nucleotide *β* at position *κ* of the PWM, *N*(*β*,*κ*) is the count of nucleotide *β* at position *κ* in the training sample.

The sum of the positional weights for a site yields the site score:
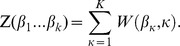



Sliding window average score (SWAS) plots were constructed as follows. The upstream regions of each gene from the CytR regulon were aligned in the bacterial groups with approximately constant CRP-CRP distance. Within a sliding window of size 8 nt, the average score of sites within the window was calculated using respective PWMs. Scores of sequences containing gaps in a given window position were set to 0, but these sequences were counted for averaging; hence, positions with gaps were penalized.

The positional information content was calculated as
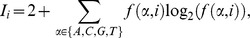
where ƒ(α, *i*) is the frequency of nucleotide α in the alignment position *i*.

## Supporting Information

Figure S1 **Alignment and SWAS plots of upstream regions of** ***deoC*** **in close relatives of** ***E. coli*** **.** Notation as in Fig. 5.
(TIF)

Figure S2 **Alignment and SWAS plots of upstream regions of** ***deoC*** **in distant Enterobacteriales.** Notation as in Fig. 5.
(TIF)

Figure S3 **Alignment and SWAS plots of upstream regions of** ***ppiA*** **in close relatives of** ***E. coli*** **.** Notation as in Fig. 5.
(TIF)

Figure S4 **Alignment and SWAS plots of upstream regions of** ***rpoH*** **in close relatives of** ***E. coli*** **.** Notation as in Fig. 5.
(TIF)

Figure S5 **Alignment and SWAS plots of upstream regions of** ***tsx*** **in distant Enterobacteriales.** Notation as in Fig. 5.
(TIF)

Figure S6 **Alignment and SWAS plots of upstream regions of** ***tsx*** **in the Vibrionales.** Notation as in Fig. 5.
(TIF)

Figure S7 **Alignment and SWAS plots of upstream regions of** ***cdd*** **in distant Enterobacteriales (direct O_CYTR_ repeats).** Notation as in Fig. 5.
(TIF)

Figure S8 **Alignment and SWAS plots of upstream regions of** ***cdd*** **in the Vibrionales.** Notation as in Fig. 5.
(TIF)

Figure S9 **Alignment and SWAS plots of upstream regions of** ***nupC-*** **in close relatives of** ***E. coli*** **(direct O_CYTR_ repeats).** Notation as in Fig. 5.
(TIF)

Table S1 **The list of orthologs of CytR-regulated genes from** ***E.coli*** **that have nearly constant O_CRP_-O_CRP_ distances.** & – “yes(value)”: the corresponding ortholog exists and the value in parentheses is the O_CRP_-O_CRP_ distance && – 0: no ortholog. # – “no(abbreviation)”, the reason why the upstream region was not considered: 1 – (single) for *cytR* means only one O_CRP_; 2 – (pstn), atypically distant start position for the group; 3 - (wght): weak maximal weight of the O_CRP_-O_CRP_ operator pair; 4 - (dist): distance larger and smaller than ±2 nucleotides compared with the average distance for the group; 5 - (mist): probable misannotation (*nupG* in *Salmonella enteric* Heidelberg and *cytR* in *Edwardsiella ictaluri*) 6 - (triple): no triple O_CRP_-O_CRP_-O_CRP_ in the specified region. @ – IIc: the second chromosomes for all *Vibrio* –spp. and Photobacterium profundum * – candidate member of the CytR regulon ** – exceptional genes added as known from the literature co – cut-off, the smallest score of known cassettes for the respective gene.
(TIF)

Table S2 **O_CRP_D-O_CYTR_D-O_CYTR_P-O_CRP_P cassettes of probable CytR regulon members in** ***E. coli*** **.** 1 – O_CRP_D is the distal CRP-operator with respect to the transcription start. 2 – O_CYTR_D is the distal CytR-operator with respect to the transcription start. 3 – O_CYTR_P is the proximal CytR-operator with respect to the transcription start. 4 – O_CRP_P is the proximal CRP-operator with respect to the transcription start. 5 – *spr* is spacer length. 6 – *Σscore* is total score of the cassette. 7 – site score is in parentheses. $ – *start pos* is the start position of the cassette in the respective upsteam region. **@** – no direct repeats for O_CyTR_D-O_CRP_P, only inverted ones; * – known regulon member with experimentally determined cassette; # – predicted regulon member, predicted cassette; #* – known regulon member, predicted cassette.
(TIF)
