# Supplementary figures and images for: Comparative Genomics of CytR, an Unusual Member of the LacI Family of Transcription Factors
Source: PLoS One. 2012 Sep 24;7(9):e44194. doi: 10.1371/journal.pone.0044194 (PMC3454398; doi:10.1371/journal.pone.0044194)

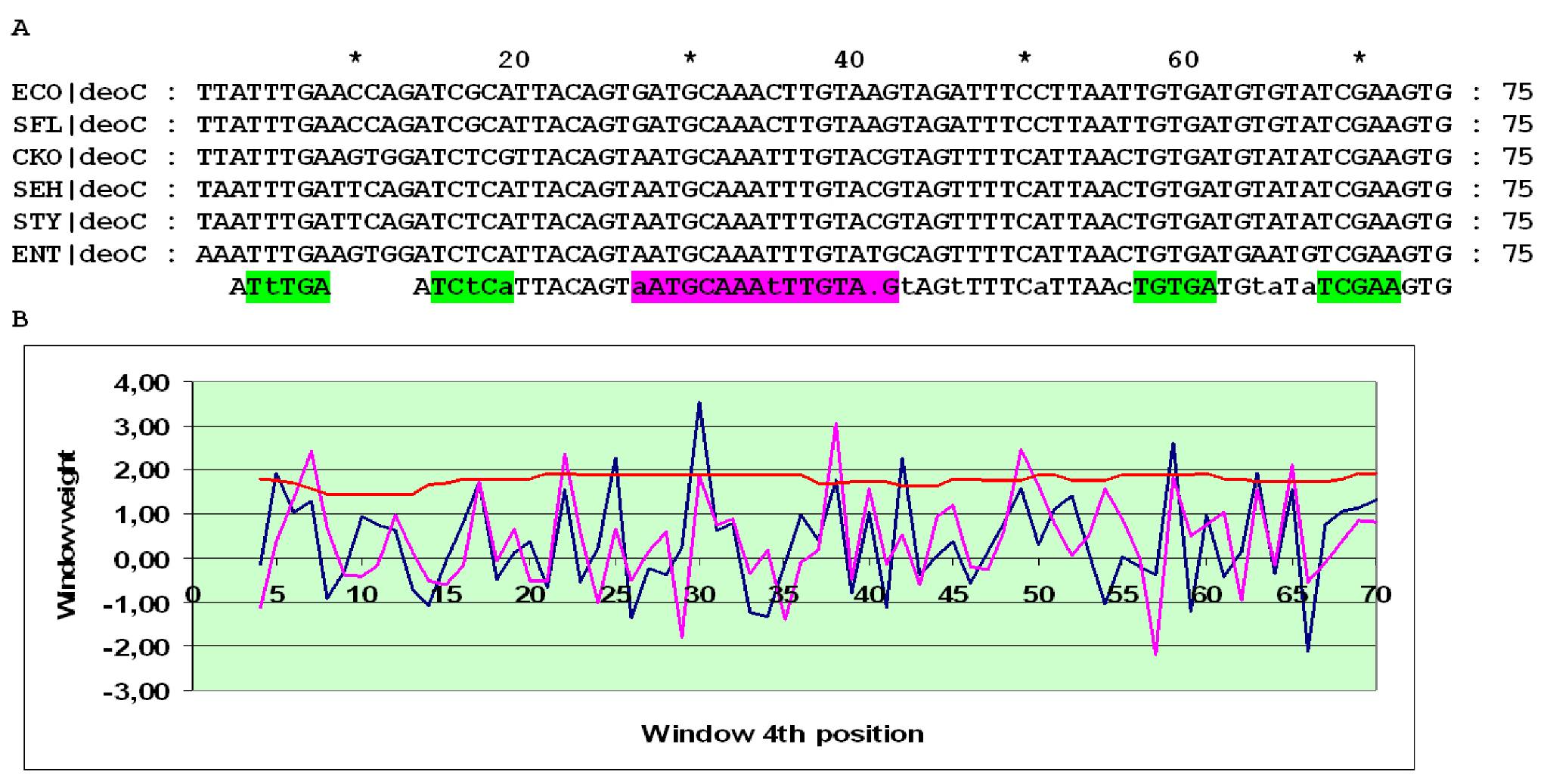

Supplement: Figure S1 — Alignment and SWAS plots of upstream regions of deoC in close relatives of E. coli . Notation as in Fig. 5. (TIF) [file pone.0044194.s001.tif]

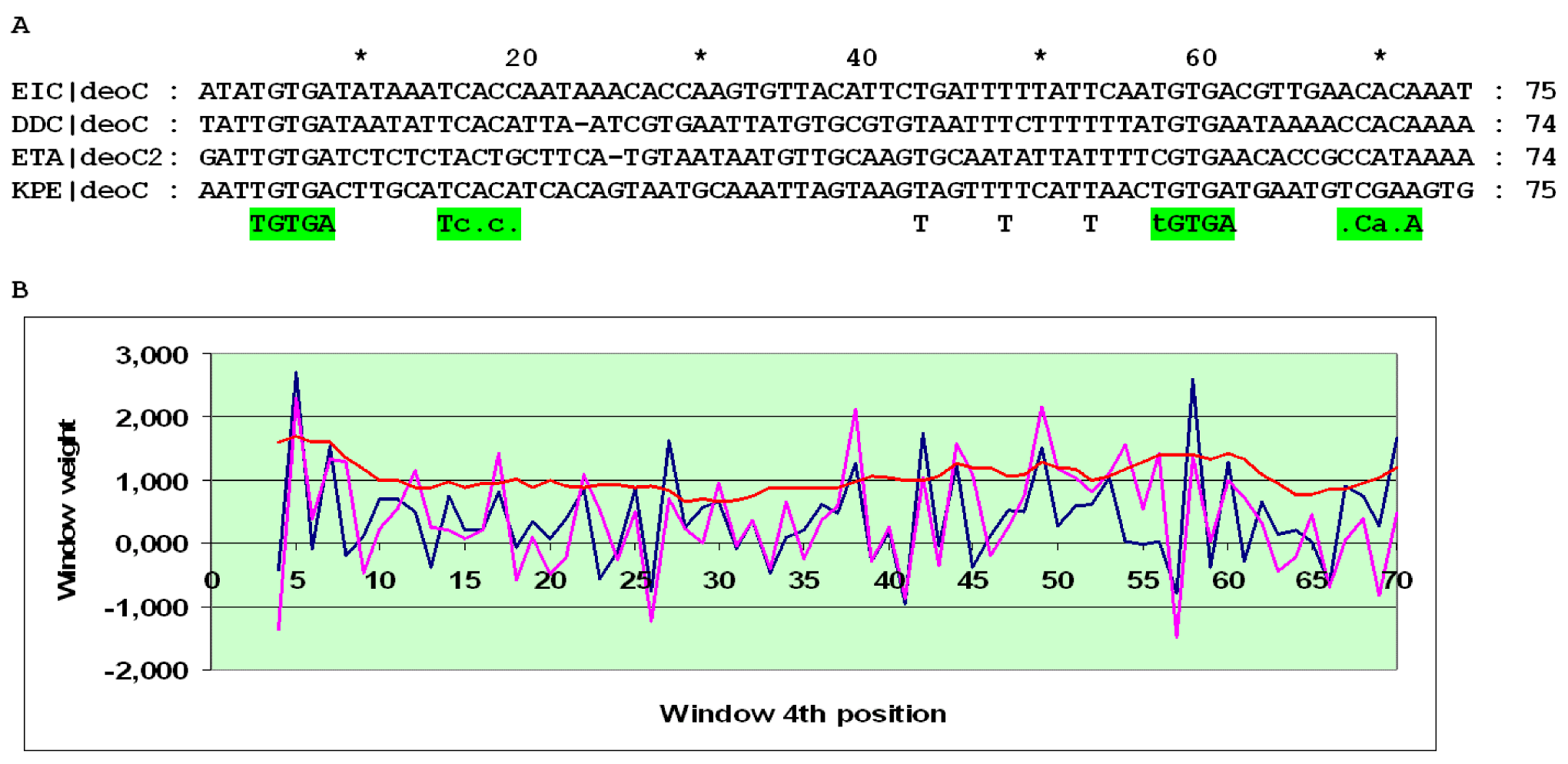

Supplement: Figure S2 — Alignment and SWAS plots of upstream regions of deoC in distant Enterobacteriales. Notation as in Fig. 5. (TIF) [file pone.0044194.s002.tif]

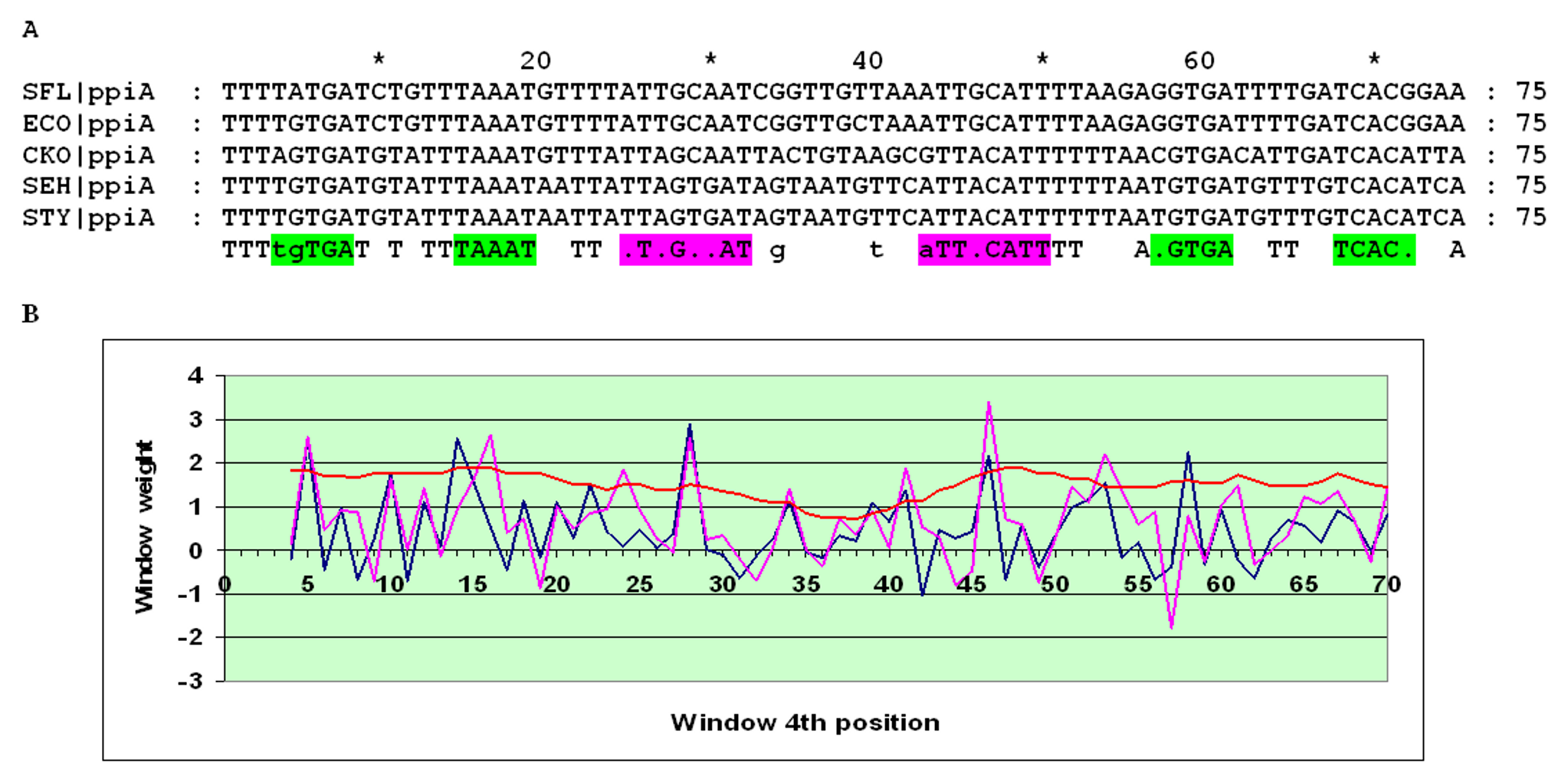

Supplement: Figure S3 — Alignment and SWAS plots of upstream regions of ppiA in close relatives of E. coli . Notation as in Fig. 5. (TIF) [file pone.0044194.s003.tif]

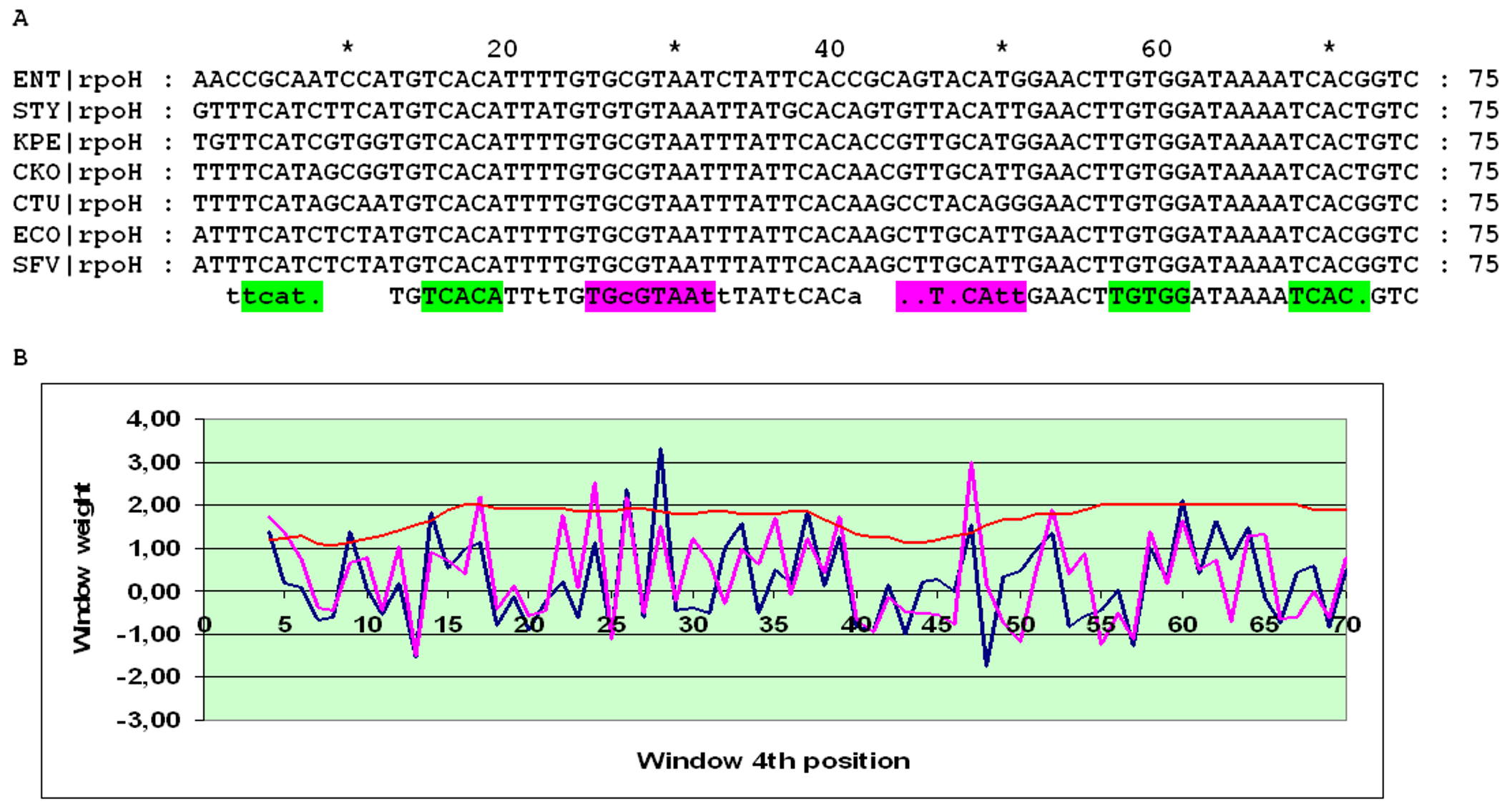

Supplement: Figure S4 — Alignment and SWAS plots of upstream regions of rpoH in close relatives of E. coli . Notation as in Fig. 5. (TIF) [file pone.0044194.s004.tif]

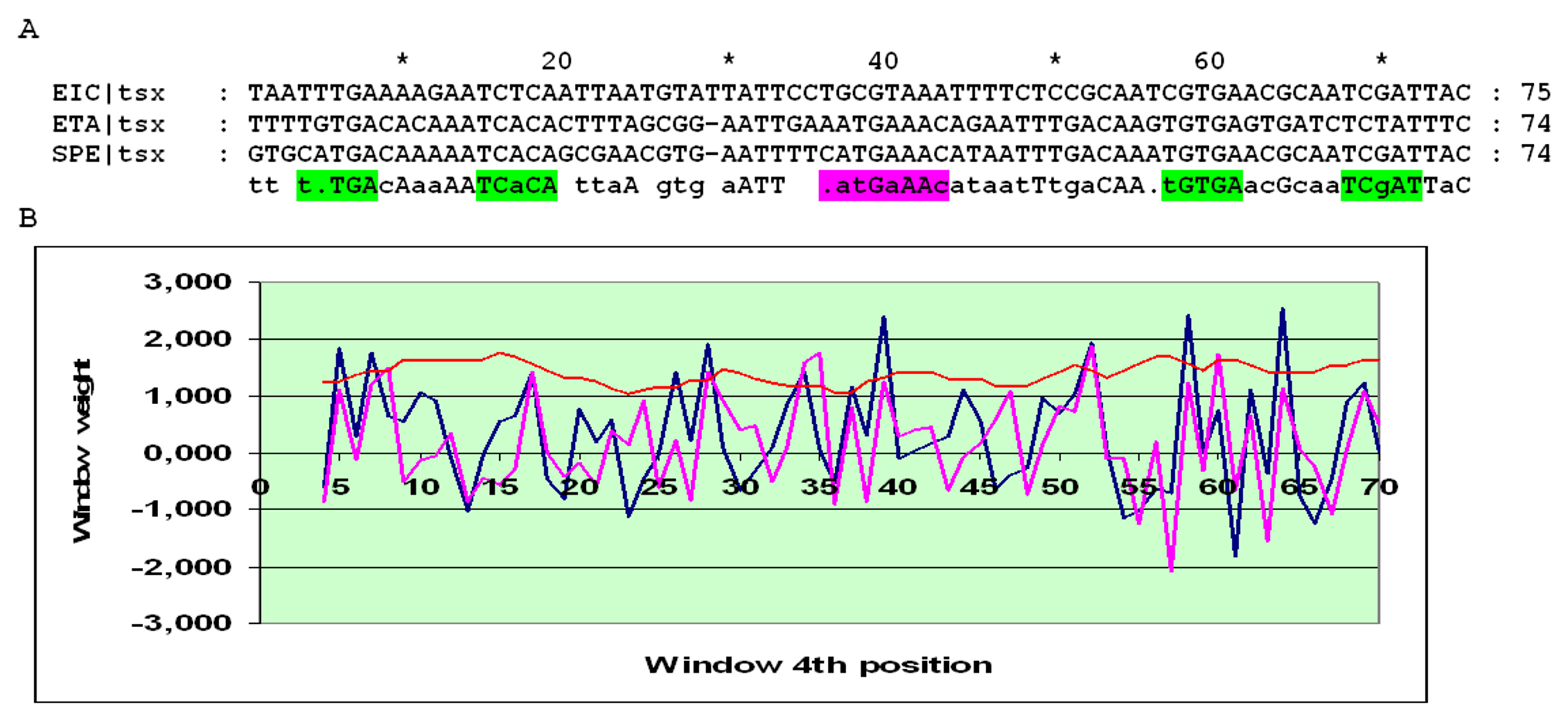

Supplement: Figure S5 — Alignment and SWAS plots of upstream regions of tsx in distant Enterobacteriales. Notation as in Fig. 5. (TIF) [file pone.0044194.s005.tif]

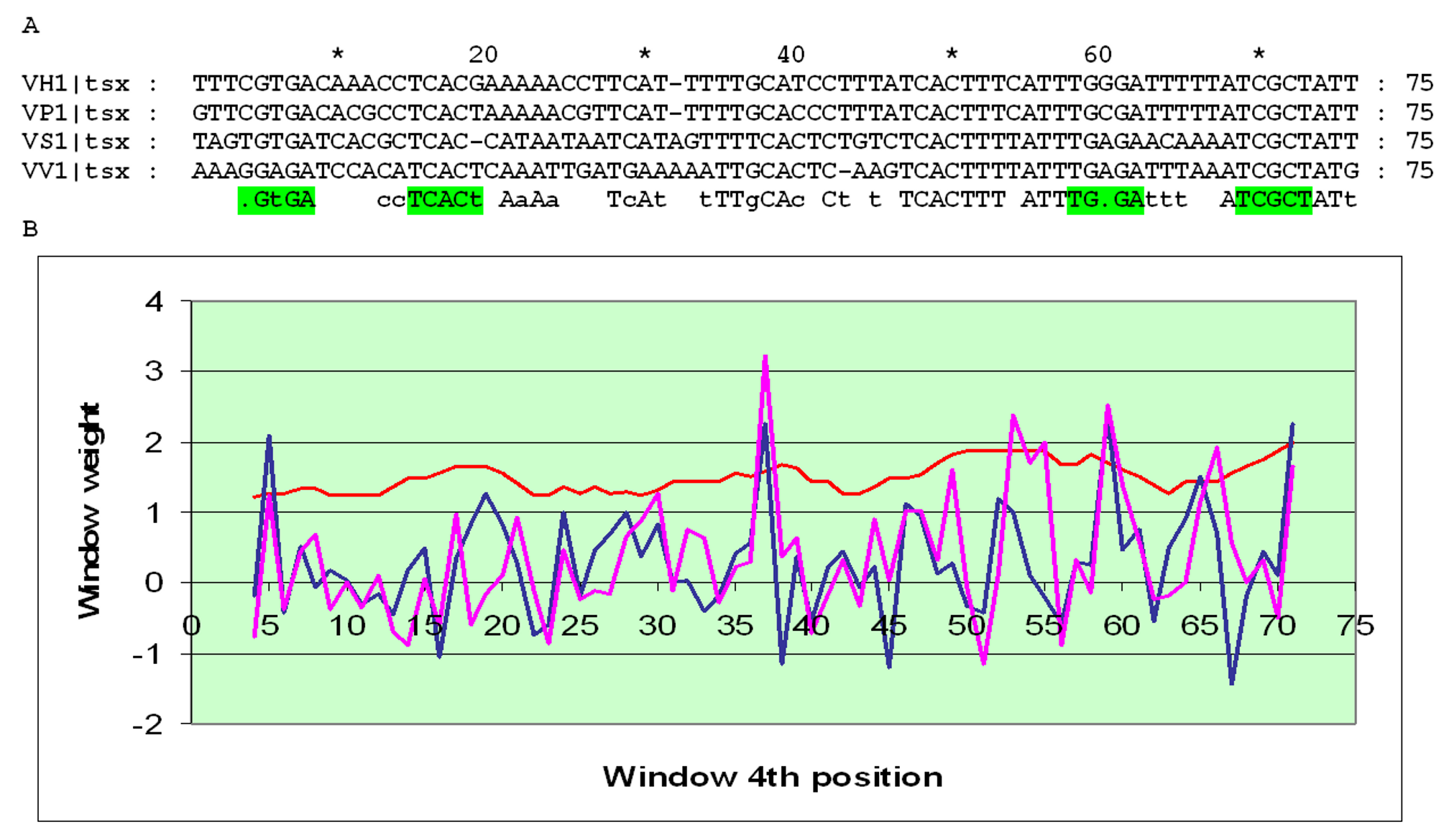

Supplement: Figure S6 — Alignment and SWAS plots of upstream regions of tsx in the Vibrionales. Notation as in Fig. 5. (TIF) [file pone.0044194.s006.tif]

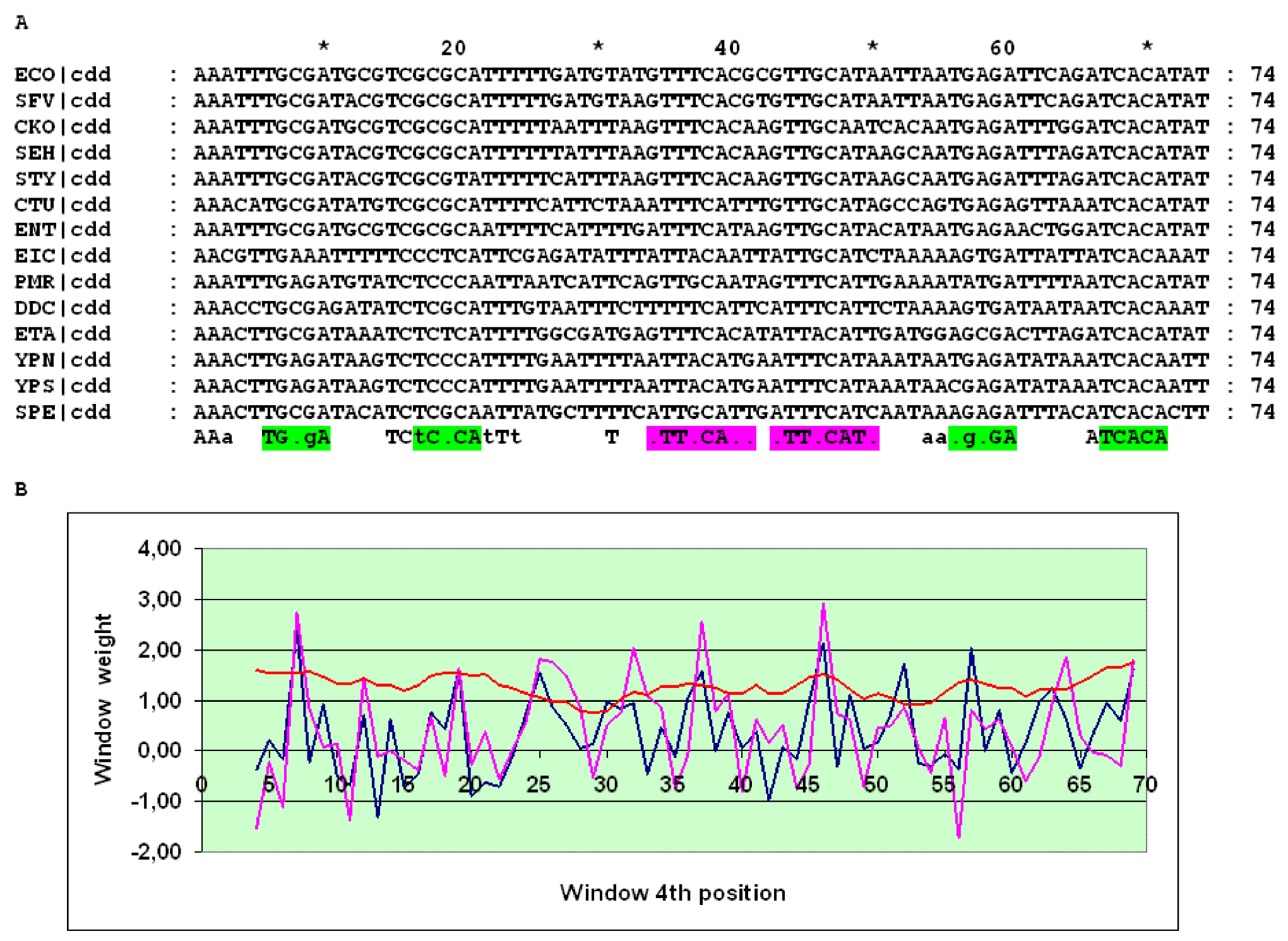

Supplement: Figure S7 — Alignment and SWAS plots of upstream regions of cdd in distant Enterobacteriales (direct OCYTR repeats). Notation as in Fig. 5. (TIF) [file pone.0044194.s007.tif]

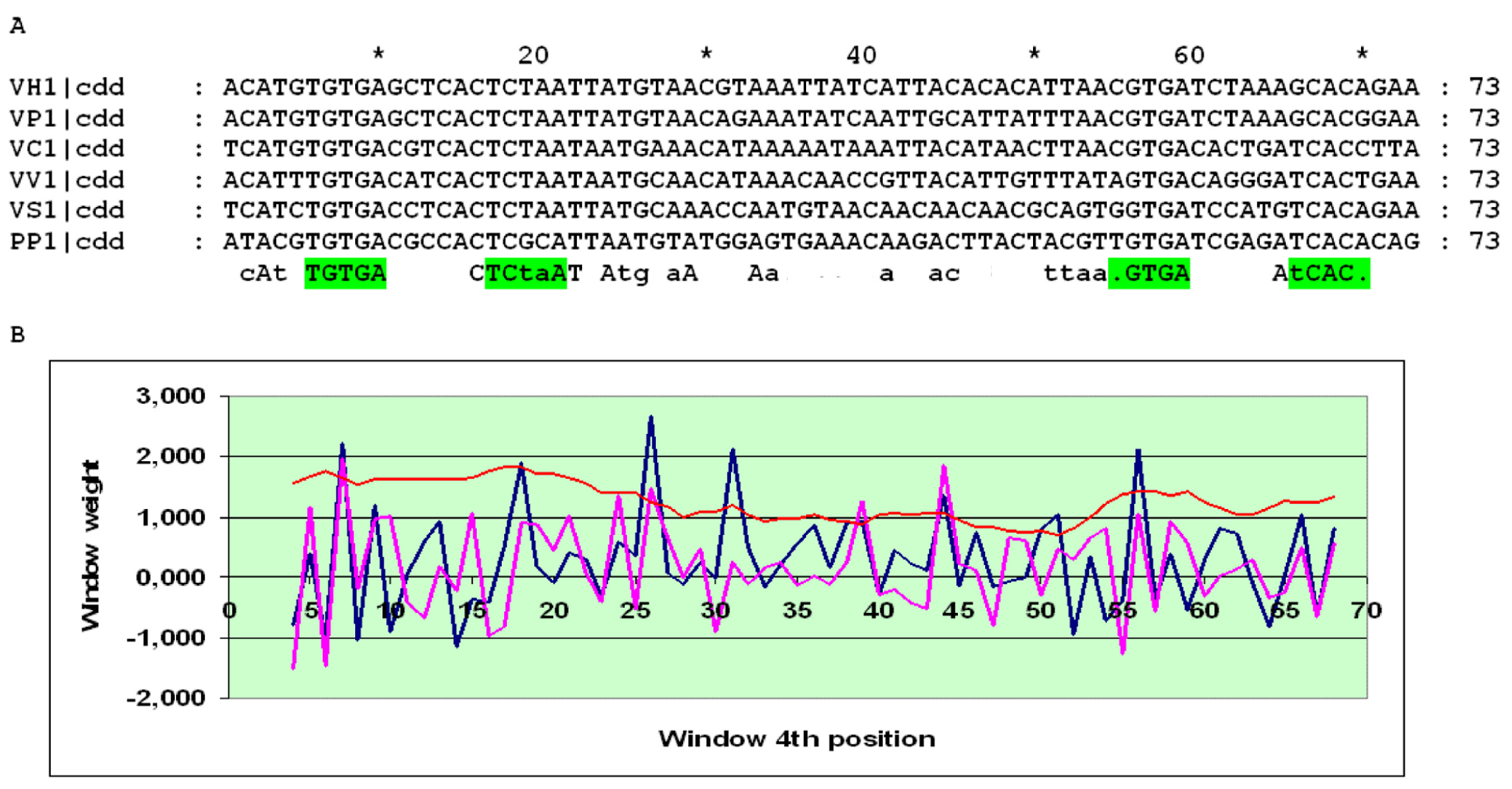

Supplement: Figure S8 — Alignment and SWAS plots of upstream regions of cdd in the Vibrionales. Notation as in Fig. 5. (TIF) [file pone.0044194.s008.tif]

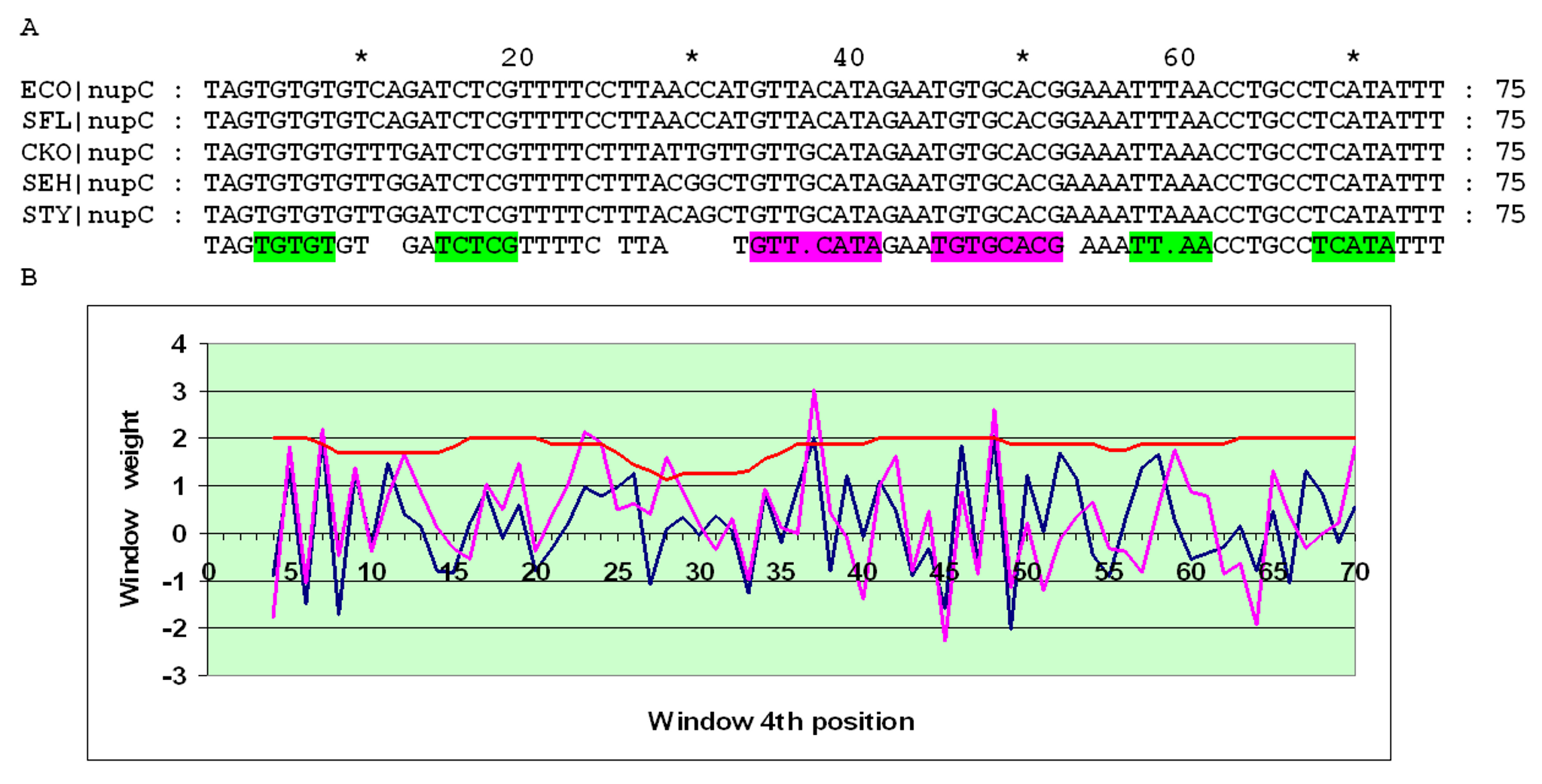

Supplement: Figure S9 — Alignment and SWAS plots of upstream regions of nupC- in close relatives of E. coli (direct OCYTR repeats). Notation as in Fig. 5. (TIF) [file pone.0044194.s009.tif]

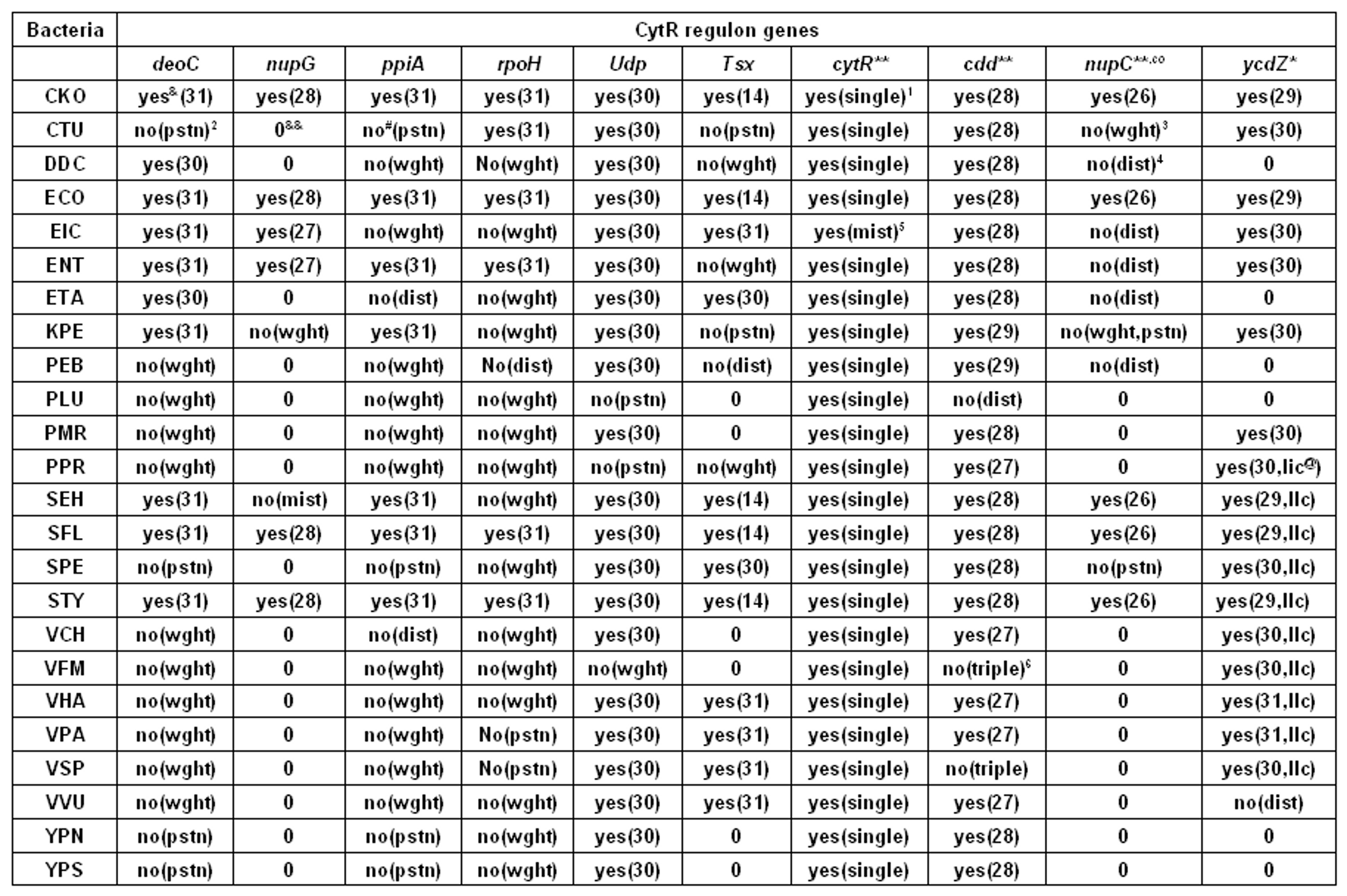

Supplement: Table S1 — The list of orthologs of CytR-regulated genes from E.coli that have nearly constant OCRP-OCRP distances. & – “yes(value)”: the corresponding ortholog exists and the value in parentheses is the OCRP-OCRP distance && – 0: no ortholog. # – “no(abbreviation)”, the reason why the upstream region was not considered: 1 – (single) for cytR means only one OCRP; 2 – (pstn), atypically distant start position for the group; 3 - (wght): weak maximal weight of the OCRP-OCRP operator pair; 4 - (dist): distance larger and smaller than ±2 nucleotides compared with the average distance for the group; 5 - (mist): probable misannotation (nupG in Salmonella enteric Heidelberg and cytR in Edwardsiella ictaluri) 6 - (triple): no triple OCRP-OCRP-OCRP in the specified region. @ – IIc: the second chromosomes for all Vibrio –spp. and Photobacterium profundum * – candidate member of the CytR regulon ** – exceptional genes added as known from the literature co – cut-off, the smallest score of known cassettes for the respective gene. (TIF) [file pone.0044194.s010.tif]

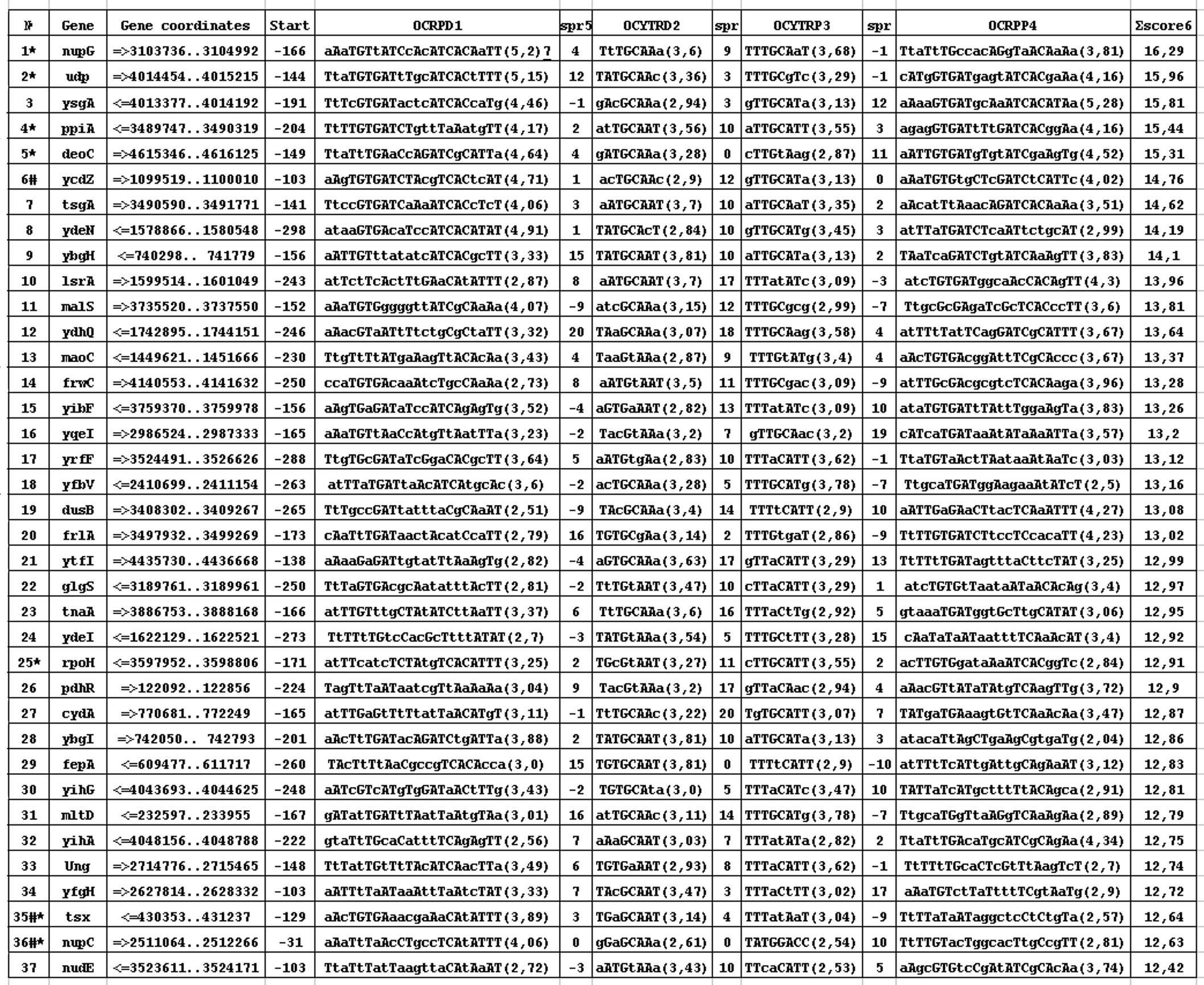

Supplement: Table S2 — OCRPD-OCYTRD-OCYTRP-OCRPP cassettes of probable CytR regulon members in E. coli . 1 – OCRPD is the distal CRP-operator with respect to the transcription start. 2 – OCYTRD is the distal CytR-operator with respect to the transcription start. 3 – OCYTRP is the proximal CytR-operator with respect to the transcription start. 4 – OCRPP is the proximal CRP-operator with respect to the transcription start. 5 – spr is spacer length. 6 – Σscore is total score of the cassette. 7 – site score is in parentheses. $ – start pos is the start position of the cassette in the respective upsteam region. @ – no direct repeats for OCyTRD-OCRPP, only inverted ones; * – known regulon member with experimentally determined cassette; # – predicted regulon member, predicted cassette; #* – known regulon member, predicted cassette. (TIF) [file pone.0044194.s011.tif]
